# Inactivation of the Thymidylate Synthase *thyA* in Non-typeable *Haemophilus influenzae* Modulates Antibiotic Resistance and Has a Strong Impact on Its Interplay with the Host Airways

**DOI:** 10.3389/fcimb.2017.00266

**Published:** 2017-06-20

**Authors:** Irene Rodríguez-Arce, Sara Martí, Begoña Euba, Ariadna Fernández-Calvet, Javier Moleres, Nahikari López-López, Montserrat Barberán, José Ramos-Vivas, Fe Tubau, Carmen Losa, Carmen Ardanuy, José Leiva, José E. Yuste, Junkal Garmendia

**Affiliations:** ^1^Instituto de Agrobiotecnología, Consejo Superior de Investigaciones Científicas-Universidad Pública Navarra-GobiernoNavarra, Spain; ^2^Centro de Investigación Biomédica en Red de Enfermedades RespiratoriasMadrid, Spain; ^3^Departamento Microbiología, Hospital Universitari Bellvitge, University of Barcelona, Institut d'Investigació Biomédica de BellvitgeBarcelona, Spain; ^4^Facultad de Veterinaria, Universidad de ZaragozaZaragoza, Spain; ^5^Servicio Microbiología, Hospital Universitario Marqués de Valdecilla and Instituto de Investigación Marqués de ValdecillaSantander, Spain; ^6^Red Española de Investigación en Patología Infecciosa, Instituto de Salud Carlos IIIMadrid, Spain; ^7^Servicio de Microbiología, Clínica Universidad de NavarraNavarra, Spain; ^8^Centro Nacional de Microbiología, Instituto de Salud Carlos IIIMadrid, Spain

**Keywords:** *Haemophilus influenzae*, thymidylate synthase, thymidine auxotrophy, thymidine uptake, bacterial morphology, antibiotic resistance, airway infection

## Abstract

Antibacterial treatment with cotrimoxazol (TxS), a combination of trimethoprim and sulfamethoxazole, generates resistance by, among others, acquisition of thymidine auxotrophy associated with mutations in the thymidylate synthase gene *thyA*, which can modify the biology of infection. The opportunistic pathogen non-typeable *Haemophilus influenzae* (NTHi) is frequently encountered in the lower airways of chronic obstructive pulmonary disease (COPD) patients, and associated with acute exacerbation of COPD symptoms. Increasing resistance of NTHi to TxS limits its suitability as initial antibacterial against COPD exacerbation, although its relationship with thymidine auxotrophy is unknown. In this study, the analysis of 2,542 NTHi isolates recovered at Bellvitge University Hospital (Spain) in the period 2010–2014 revealed 119 strains forming slow-growing colonies on the thymidine low concentration medium Mueller Hinton Fastidious, including one strain isolated from a COPD patient undergoing TxS therapy that was a reversible thymidine auxotroph. To assess the impact of thymidine auxotrophy in the NTHi-host interplay during respiratory infection, *thyA* mutants were generated in both the clinical isolate NTHi375 and the reference strain RdKW20. Inactivation of the *thyA* gene increased TxS resistance, but also promoted morphological changes consistent with elongation and impaired bacterial division, which altered *H. influenzae* self-aggregation, phosphorylcholine level, C3b deposition, and airway epithelial infection patterns. Availability of external thymidine contributed to overcome such auxotrophy and TxS effect, potentially facilitated by the nucleoside transporter *nupC*. Although, *thyA* inactivation resulted in bacterial attenuation in a lung infection mouse model, it also rendered a lower clearance upon a TxS challenge *in vivo*. Thus, our results show that thymidine auxotrophy modulates both the NTHi host airway interplay and antibiotic resistance, which should be considered at the clinical setting for the consequences of TxS administration.

## Introduction

Non-typeable (non-capsulated) *Haemophilus influenzae* (NTHi) is a Gram negative coccobacillus that is a common commensal in the nasopharynx of healthy humans, and also an opportunistic pathogen causing respiratory infections such as acute otitis media, otitis media with effusion, community-acquired pneumonia, and exacerbations of chronic obstructive pulmonary disease (COPD; Agrawal and Murphy, 2011). COPD is characterized by a progressive and not fully reversible airflow limitation, accompanied by infiltration of the airways by neutrophils and mucus hypersecretion (Barnes, 2016). The chronic course and evolution of COPD is often characterized by periods of symptom exacerbation with a negative impact on the patient's quality of life and evolution of the disease, and represent a significant cause of medical intervention and hospitalization. Given that acute exacerbations of COPD (AECOPD) are mostly caused by bacterial and viral infections (Sethi, 2010), antibiotic therapy is routinely prescribed. Use of amoxicillin, tetracyclines, or cotrimoxazole (TxS), a combination of trimethoprim (TMP) and sulfamethoxazole (SMX), has long been regarded as standard therapy for patients with AECOPD, which has contributed to the increasing emergence of resistance to these antibiotics in common respiratory pathogens such as NTHi, and become a real challenge in the choice of adequate antibacterials (Nouira et al., 2010).

The treatment of respiratory infections has been a very important field of use of TxS, as a first-line agent against *H. influenzae* infected respiratory patients (Iyer Parameswaran and Murphy, 2009), and has also been considered as a prophylactic option in HIV-infected children therefore having an impact on *H. influenzae* carriage (Grant et al., 2009; Mwenya et al., 2010), altogether increasing TxS resistance levels in this pathogen. TxS interferes with the bacterial metabolism and replication by blocking the production of tetrahydrofolic acid (THF). THF is a co-factor for the thymidylate synthase, an essential protein encoded by the *thyA* gene, required for the conversion of thymidine from uracil (Stryer, 1995). During normal metabolism, dihydrofolate is reduced to THF by the dihydrofolate reductase (DHFR), encoded by the *folH* gene, also known as *drfA* and *folA*. TMP is a substrate analog of dihydrofolate and blocks its reduction to THF. Conversely, SMX is a substrate analog of *para*-aminobenzoic acid, and blocks the *folP* gene encoding the dihydropteroate synthetase DHPS, which is involved in production of dihydropteroate, a precursor of dihydrofolate. Both TMP and SMX have little toxicity to humans because humans do not synthesize folic acid but obtain it from dietary sources (Tristram et al., 2007). Inhibiting the production of THF prevents thymine synthesis and, hence, DNA replication, causing bacterial death and TxS susceptibility. However, if external thymidine is available, as shown in infected tissues (Besier et al., 2008), thymidine-dependent TxS resistance may emerge upon treatment. This aspect has been long reported (Maskell et al., 1978), and extensively analyzed in *Staphylococcus aureus*, where the underlying mechanism for thymidine dependency relies on mutations of the *thyA* gene. Thus, inactivation of the *thyA* gene generates thymidine auxotroph small colony variants (SCVs) with a strong impact in this pathogen's physiology, virulence, and persistence, and uptake of external thymidine by the *S. aureus* NupC nucleoside transporter seems to bypass the effect of TxS (Kriegeskorte et al., 2014).

In NTHi, resistance to TxS is associated to polymorphisms and/or short insertions in the *folH* and *folP* genes, DHFR overproduction, or acquisition of the sulfonamide (SUL) genes *sul1* and *sul2* (de Groot et al., 1988, 1996; Enne et al., 2002). Existing evidence also relates *H. influenzae* TMP resistance to transient thymidine auxotrophy in isolates from sputum samples of chronic bronchitis patients receiving TMP (Platt et al., 1983). Despite this observation, little is known about thymidine-dependent antibiotic resistance and its underlying consequences for NTHi pathogenesis. Following this notion, we observed that among 2,542 NTHi strains isolated between 2010 and 2014 from clinical samples at Bellvitge University Hospital (Spain), 119 strains formed slow-growing colonies on Mueller Hinton Fastidious (MH-F) agar, a thymidine low concentration medium. This observation prompted us to hypothesize that such slow-growth could relate to thymidine auxotrophy, rendering TxS resistance due to the antibiotic administration. If so, such auxotrophy could also modify the dynamics of the NTHi-host interplay. To address these hypotheses, we screened the available NTHi isolates with a slow growth on MH-F agar and identified one thymidine auxotroph, easily reversible to the normal phenotype. Moreover, to question the relationship between thymidine auxotrophy and respiratory infection by NTHi, we employed two genome sequenced strains, NTHi strain 375, hereafter NTHi375, and *H. influenzae* (Hi) RdKW20 (Fleischmann et al., 1995; Mell et al., 2014), to generate thymidine auxotrophs by mutating the *thyA* gene, and systematically evaluated its effect on (i) bacterial resistance to TxS, morphology, growth, self-aggregation, and gene expression; (ii) NTHi-host interplay by assessing bacterial binding to a panel of components of the complement system, adhesion to- and invasion of cultured airway epithelia; (iii) NTHi respiratory infection *in vivo* by using a murine intranasal infection model upon TxS treatment. This work provides further evidence of the emergence of NTHi TxS resistance upon its administration, and shows for the first time that mutation of the thymidylate synthase encoding gene *thyA* in NTHi leads to a strong impact on its physiology and virulence, but also provides a survival advantage during TxS challenge. Our results provide a context for a better understanding of the potential effects of TxS treatment against NTHi respiratory infection.

## Materials and methods

### Bacterial strains and growth conditions

Strains used in this study are described in Table 1. NTHi strains were grown at 37°C, 5% CO_2_ on chocolate agar (Biomérieux), MH-F agar (Biomérieux), or brain-heart infusion (BHI) agar supplemented with 10 μg/ml hemin and 10 μg/ml nicotinamide adenine dinucleotide (NAD), referred to as sBHI. NTHi liquid cultures were grown in sBHI (37°C, 5% CO_2_). Thymidine (Sigma-Aldrich) was dissolved in sterile distilled water (stock solution, 10 mg/ml). When necessary, media were supplemented with thymidine by (i) using sterile paper discs soaked on thymidine 300 μg/ml or 10 mg/ml, (ii) thymidine 300 μg/ml spreading on 20 ml chocolate agar or MH-F agar plates, (iii) thymidine 300 μg/ml addition into sBHI. Erythromycin 11 μg/ml (Erm_11_) or spectinomycin 50 μg/ml (Spec_50_) were used when required. *Escherichia coli* was grown on Luria Bertani (LB) agar at 37°C, supplemented with ampicillin 100 μg/ml (Amp_100_), erythromycin 150 μg/ml (Erm_150_) or Spec_50_, when necessary.

**Table 1:** Strains and plasmids used in this study.

	**Description**	**Source**
**STRAINS**		
***Haemophilus influenzae***		
NTHi375	Wild-type, otitis media clinical isolate	Hood et al., 1999
NTHi375Δ*thyA*	*thyA::ermC*, Erm^R^	This study
NTHi375Δ*nupC*	*nupC::spec*, Spec^R^	This study
RdKW20	Laboratory strain, capsule-deficient serotype d	Fleischmann et al., 1995
RdKW20Δ*thyA*	*thyA::ermC*, Erm^R^	This study
RdKW20Δ*nupC*	*nupC::spec*, Spec^R^	This study
NTHi8233	COPD isolate from a sputum sample, thymidine auxotroph	This study
***Escherichia coli***		
TOP 10	Used for cloning assays	Thermofisher scientific
SW102	Derived from DY380, it contains a defective λ prophage with the recombination proteins *exo, bet*, and *gam* being controlled by the temperature-sensitive repressor *cI*857	Tracy et al., 2008
**PLASMIDS**		
pJET1.2	Cloning vector	Thermofisher scientific
pBSLerm	Source of an Erm^R^ cassette	Allen et al., 2005
pJET1.2-*thyA*	pJET1.2 with a 2,852 bp insert containing the *thyA*_NTHi375_ gene (852 bp) and 1 kb flanking regions	This study
pJET1.2-*thyA::ermC*	pJET1.2 with a 3,814 bp insert containing a *thyA*_NTHi375_*::ermC* disruption cassette	This study
pJET1.2-*nupC*	pJET1.2 with a 2,664 bp insert containing the *nupC*_RdKW20_ gene (1,254 bp) gene and ~700 bp flanking regions	This study
pJET1.2-*nupC::spec*	pJET1.2 with a 3,501 bp insert containing a *nupC*_RdKW20_*::spec* disruption cassette	This study
pRSM2832	pKD13 derivative carrying a cassette containing a spectinomycin resistance gene flanked by FRT sites	Tracy et al., 2008

For *thyA* disruption, a DNA fragment containing the *thyA* gene and its respective adjacent regions (2,852 bp), was PCR amplified with *Phusion* polymerase (Thermofisher) using NTHi375 genomic DNA as template and primers thyA-F1 (5′-TGCCTGAATATTCGCTCGGTTACATTTA) and thyA-R1 (5′-CGATGATACTTAAAAGTAATCGCGACCAAAAATTCGG). The gene-containing fragment was cloned into pJET1.2 (Thermofisher), generating pJET1.2-*thyA*. This cloned PCR product was disrupted by inverse PCR with *Phusion* polymerase, using primers thyA-F2 (5′-CGTTCCTGTGATGTTCCGCTTGGA) and thyA-R2 (5′-ATATCATCAACGCCTCTACGATGC). An internal 226-bp fragment (nucleotides 312–537 in the *thyA* coding sequence) was replaced by a blunt-ended erythromycin resistance cassette excised by *Sma*I digestion from pBSLerm (Allen et al., 2005), generating pJET1.2-*thyA::ermC*. This plasmid was used as a template to amplify the *thyA::ermC* disruption cassette with primers thyA-F1 and thyA-R1, which was used to transform NTHi375 using the MIV method (Herriott et al., 1970). Transformants were screened by plating bacteria on sBHI agar with Erm_11_ to obtain NTHi375Δ*thyA*. Same approach and disruption cassette were used to generate RdKW20Δ*thyA*. For mutant confirmation, NTHi375Δ*thyA* and RdKW20Δ*thyA* genomic DNA were used as template to be PCR amplified with four primer pairs: (i) thyA-F1 and thyA-R1, rendering a 3,814 bp product; (ii) thyA-F1 and pBSLerm-down (5′-GGTACACGAAAAACAAGTTAAGGG), rendering a 2,421 bp product; (iii) pBSLerm-up (5′-ATAAAGAGGGTTATAATGAACGAG) and thyA-R1, rendering a 2,190 bp product; (iv) pBSLerm-up and pBSLerm-down, rendering a 797 bp product (data not shown). Recombination events resulting from integration of the *thyA* disruption cassette in the NTHi375 and RdKW20 genomes were further verified by NTHi375Δ*thyA* and RdKW20Δ*thyA* genomic DNA PCR amplification with primers thyA-F1 and thyA-R1, and PCR product sequencing with primers thyA-F1, pBSLerm-up, pBSLerm-down and thyA-R1. When necessary, the *thyA* gene was PCR amplified with primers ThyA_Pro_F (5′-TGCGCCTTTGATTCCGTTTG) and ThyA_R2 (5′-TCACCTAACGCTTCCGCTTT) for DNA sequencing.

The *nupC* gene and its respective adjacent regions (2,664 bp) was amplified by PCR with *Phusion* polymerase using RdKW20 genomic DNA as template and primers nupC-F1 (5′-ATGAACAGGTTATGGAGGCAGTTCCAT) and nupC-R1 (5′-GTGAGTACGAATATGGTCAGACACGGT). The gene-containing fragment was cloned into pJET1.2, generating pJET1.2-*nupC*. A Spec^r^ cassette was PCR amplified from pRSM2832 using gene-specific mutagenic primers nupC-F2 (5′-GTAGAATAAGCCGAATTTTATTAACTTAACTAATCTAGGGGAATCAAATGATTCCGGGGATCCGTCGACC) and nupC-R2 (5′-TACCGCACTTTTTAATTGATTAAGATAAATTAAAGTGCTGCAGCACCTAAGCCTGTAGGCTGGAGCTGCTTCG), as described previously (Tracy et al., 2008). Primers were designed to delete sequences between the start codon and the last seven codons of *nupC*. *E. coli* SW102 cells were prepared for recombineering, co-electroporated with pJET1.2-*nupC* (Amp^r^; 50 ng) and the *nupC*-specific mutagenic cassette (Spec^r^; 200 ng) as previously described (Sinha et al., 2012), and mutagenized clones containing pJET1.2-*nupC::spec* were selected on LB agar with Amp_100_, Spec_50_. This plasmid was used as a template to amplify the *nupC::spec* disruption cassette with primers nupC-F1 and nupC-R1, which was used to transform NTHi375 and RdKW20 using the MIV method. Transformants were selected on sBHI agar with Spec_50_, to obtain NTHi375Δ*nupC* and RdKW20Δ*nupC* mutant strains.

### Screening for NTHi thymidine auxotroph clinical isolates

Laboratory records of Microbiology Department at the Bellvitge University Hospital (Spain) regarding the difficulties of some NTHi isolates to growth on conventional antimicrobial susceptibility testing medium (Mueller-Hinton agar+5% defibrinated horse blood and 20 mg/l β-NAD, MH-F) were reviewed from 2010 to 2014. Screening for thymidine auxotrophs among NTHi clinical isolates was based on the interpretation of their growth characteristics. Isolates identified as having growth problems were further screened for thymidine auxotrophy by comparative growth on chocolate agar plates and low-thymidine MH-F agar plates. Thymidine auxotrophy was confirmed by testing strain growth on MH-F agar in the absence or presence of discs soaked with thymidine.

### Susceptibility testing under non-standard conditions

TxS susceptibility testing of NTHi thymidine auxotrophs failed to produce results by disc diffusion (Becton Dickinson), *E*-test (Biomérieux) or broth microdilution when inoculated on MH-F and incubated for 24 h, as specified by the European Committee on Antimicrobial Susceptibility Testing (EUCAST; http://www.eucast.org/clinical_breakpoints). Given that chocolate agar supported growth for all strains after 24 h of incubation, this medium was used for *E*-test- or TxS discs-based determination of minimal inhibitory concentrations (MIC). Strains were grown on chocolate agar, to generate bacterial suspensions normalized in phosphate-buffered saline (PBS) to OD_600_ = 1. Normalized suspensions were spread on chocolate agar or, when necessary, on MH-F agar, in the presence of *E*-test or TxS discs (23.75 mg/1.25 mg SMX:TMP) and incubated for 24 h before assessing the diameter of the growth inhibition zones.

### Growth curves

To monitor growth, NTHi strains grown on chocolate agar for 16 h were inoculated (2–5 colonies) in 20 ml sBHI, with or without thymidine, and incubated for 11 h with shaking. Cultures were diluted in 40 ml sBHI, with or without thymidine, to OD_600_ = 0.01 (RdKW20) or OD_600_ = 0.05 (NTHi375), incubated with agitation, and OD_600_ was recorded every hour for 8 h. Every 2 h, culture samples were serially diluted and plated on sBHI agar. Data are shown both as OD_600_ and c.f.u./ml. At the final time point, 10 μl of the bacterial cultures were placed on glass coverslips and fixed with 3.7% paraformaldehyde (PFA) in PBS pH 7.4 for 15 min at room temperature. Bacteria were labeled with a polyclonal rabbit anti-NTHi primary antibody diluted 1:600 and a donkey anti-rabbit conjugated to Cy2 (Jackson Immunological) secondary antibody diluted 1:100. Samples were analyzed with a Carl Zeiss Axioskop 2 plus fluorescence microscope and a Carl Zeiss Axio Cam MRm monochrome camera.

### Confocal microscopy

NTHi strains were grown on chocolate agar for 16 h, in the absence or presence of thymidine, and a colony was aseptically spread with an inoculation loop over a drop of distilled water on a microscopy slide. Samples were air-dried and stained for 15 min in the darkness with the cell-permeable fluorescent nucleic acid stain SYTO 9 (Life Technologies), following the manufacturer's instructions. Samples were washed twice with distilled water and fluorescence was observed by confocal laser microscopy. Images were acquired using a Leica TCS-SL filter-free spectral confocal laser-scanning microscope (Leica Microsystems) equipped with a 488 nm argon laser, 543 nm and 633 nm He/Ne lasers (Centres Científics i Tecnològics-Campus de Bellvitge, Universitat de Barcelona, Spain) using a 63× magnification oil immersion objective (1.4 numerical aperture), and an image resolution of 1024 × 1024 pixels. Images were acquired randomly and analyzed using the Leica Confocal Software 2.5 (Leica Microsystems).

### Transmission electron microscopy (TEM)

*H. influenzae* strains were examined by TEM after growth on chocolate agar following established procedures (Remuzgo-Martinez et al., 2015). Briefly, bacteria were applied to Formvar-coated grids, air dried, negatively stained with 1% phosphotungstic acid in distilled water for 10 s, and examined with a JEM-1011 transmission electron microscope (JEOL) operating at 80 kV and equipped with an Orius SC1000 charge-coupled device (CCD) camera (Gatan).

### RNA extraction and real-time quantitative PCR (RT-qPCR) analysis

NTHi strains were grown for 16 h on chocolate agar. Bacteria (2–5 colonies) were inoculated into 20 ml sBHI, grown for 11 h, with or without thymidine, diluted into 40 ml fresh sBHI to OD_600_ = 0.05, in the absence or presence of thymidine, and grown to OD_600_ = 0.6. Bacterial total RNA was isolated using TRIzol reagent (Invitrogen). Total RNA quality was evaluated using RNA 6000 Nano LabChips (Agilent 2100 Bioanalyzer). All samples had intact 16S and 23S ribosomal RNA. Complementary DNA (cDNA) was synthesized from total RNA (1 μg) using SuperScript II Reverse Transcriptase reagents (Invitrogen). Real-time quantitative PCR was performed using Thermo Scientific Luminaris HiGreen qPCR Master Mix (Thermo Scientific) and fluorescence data were analyzed with BioRad CFX96 qPCR System (Bio-Rad). Relative quantities of mRNAs were calculated using the comparative threshold cycle (Ct) method and normalized using 16S ribosomal RNA (*16SrRNA*) as an endogenous control. Primer pairs were designed with Primer3 software: for *nupC*, nupC-RT-Fw (5′-ATGTTAATCGCATTCGTTGGTT) and nupC-RT-Rv (5′-ATTTGTCCTGCAATACCTGCTT); for *tdk*, tdk-RT-Fw (5′-TGTGCTTTGTTATGGCTTGC) and tdk-RT-Rv (5′-CTTTAATGACCTCGCCCTGA); for *16SrRNA*, 16S-Fw (5′-GGCGTTGATGACCGTGAAAC) and 16S-Rv (5′-GCCAGTAATAATCGCCCTCTTCTAG). Data are expressed as relative expression on mutant strains compared to their parental wild-type strains, considered to be 1. All measures were carried out in duplicate and at least three times (*n* ≥ 6).

### Bacterial aggregation assay

Three to four colonies of NTHi grown on chocolate agar for 16 h were inoculated into 20 ml sBHI, grown for 11 h, diluted in sBHI to OD_600_ = 1 and left standing at room temperature for 5 h (starting volume ~25 ml). The viability of each culture was tested by serial dilution and plating on sBHI agar at the beginning of each experiment (*t* = 0; data not shown). OD_600_ readings were performed at regular time intervals on 700 μl aliquots collected from the top of each bacterial suspension. At least four independent experiments (*n* ≥ 4) were performed for each strain.

### Phosphorylcholine (PCho) quantification

Three to four colonies of NTHi grown on chocolate agar for 16 h were inoculated into 20 ml sBHI, grown for 11 h, diluted into 40 ml sBHI to OD_600_ = 0.05, grown to OD_600_ = 0.6 in the absence or presence of thymidine, serially diluted, plated on sBHI agar for c.f.u. determination, and used to generate stocks stored at −80°C in sBHI with 20% glycerol as single use aliquots for further experiments. For PCho determination, ~1 × 10^7^ c.f.u. were incubated for 1 h at 37°C with TEPC-15, a mouse monoclonal antibody specific for PCho (Sigma-Aldrich) diluted 1:25 in PBS-0.05% Tween 20. Samples were washed twice with PBS-0.05% Tween 20, and incubated with a fluorescein isothiocyanate (FITC)-conjugated rabbit anti-mouse (Serotec) diluted 1:300 in PBS-0.05% Tween 20 for 30 min at 4°C under dark conditions. Bacteria were washed with PBS-0.05% Tween 20, fixed in 3% paraformaldehyde (PFA) for 2–3 min at room temperature, and analyzed on a FACSCalibur flow cytometer (BD Biosciences) using forward and side scatter parameters to gate on at least 25,000 bacteria. Results are expressed as a relative percent fluorescence index (RFI), to measure both the proportion of fluorescent bacteria positive for PCho and the intensity of fluorescence (Ramos-Sevillano et al., 2015). Assays were performed in quadruplicate in at least three independent occassions (*n* ≥ 12).

### Binding of complement factors to NTHi

Bacterial strains were grown as indicated for PCho determination. C3b deposition was analyzed as explained previously (Ramos-Sevillano et al., 2015). Briefly, a bacterial suspension containing ~1 × 10^7^ c.f.u. was opsonized with human serum diluted 1:4 in PBS-0.05% Tween 20, and detected with a FITC-conjugated polyclonal goat anti-human C3b antibody (ICN-Cappel) diluted 1:300 in PBS-0.05% Tween 20 for 30 min at 4°C under dark conditions. CRP binding was measured as previously described (Ramos-Sevillano et al., 2015), by incubating ~1 × 10^7^ c.f.u. with human serum diluted 1:4 in PBS-0.05% Tween 20 and detected with a polyclonal rabbit anti-human CRP antibody (Calbiochem) for 1 h at 37°C, followed by two washes with PBS-0.05% Tween 20. Bacterial suspensions were then incubated for 30 min with a FITC-conjugated polyclonal goat anti-rabbit antibody in PBS-0.05% Tween 20 for 1 h at 37°C. For both C3b and CRP binding, bacteria were finally washed with PBS-0.05% Tween 20, fixed in 3% PFA, and analyzed on a FACSCalibur flow cytometer as described above. Results are expressed as a RFI. Assays were performed in quadruplicate in at least three independent occassions (*n* ≥ 12).

### Cell culture and bacterial infection

A549 human alveolar basal epithelial cells (ATCC CCL-185) were maintained as described (Morey et al., 2011), seeded to 6 × 10^4^ cells/well for 32 h, and serum-starved 16 h before infection. NCI H-292 mucoepidermoid pulmonary human carcinoma epithelial cells (ATCC CRL-1848) were maintained as described (Euba et al., 2015c), and seeded to 4 × 10^5^ cells/well 16 h before infection. Adhesion and invasion assays were performed and processed as described (Morey et al., 2011; Lopez-Gomez et al., 2012; Euba et al., 2015a,c). For infection, PBS-normalized bacterial suspensions (OD_600_ = 1) were prepared by using NTHi strains grown on chocolate agar for 16 h, or in 20 ml sBHI for 11 h in the absence or presence of thymidine. A multiplicity of infection (MOI) of ~100:1 was used. To monitor adhesion, cells were infected for 30 min. Although this assay does not completely exclude a possible internalization of some bacteria, experimental conditions were previously set to mainly monitor adhesion (Morey et al., 2011; data not shown). For invasion assays, cells were incubated with bacteria for 2 h, washed three times with PBS, incubated for 1 h with RPMI 1640 medium containing 10% FCS, Hepes 10 mM, and gentamicin 200 μg/ml to kill extracellular bacteria.

When necessary, A549 cells were infected for 30 min with bacterial suspensions (OD_600_ = 1) generated with bacteria grown on chocolate agar, in the absence or presence of thymidine; infections were performed in RPMI 1640, and in the absence or presence of purified C3 (16 μg/ml), 2% C3-deficient human serum (HS), or C3-depleted HS reconstituted with purified C3. Human C3-deficient serum and human complement C3 were purchased from Sigma-Aldrich. Cells were then washed three times with PBS, lysed with 300 μl of PBS-saponin 0.025% for 10 min at room temperature, and serial dilutions were plated on sBHI agar. All infections were performed in triplicate at least three independent times (*n* ≥ 9). Results are expressed as c.f.u./ml.

### Secretion of IL-8

For A549 cell stimulation by NTHi, bacteria grown on chocolate agar, in the absence or presence of thymidine, were collected with PBS, suspensions were normalized to OD_600_ = 1, and used for a 2 h infection with a MOI of ~100:1. Cells were washed three times with PBS, and incubated for 6 h in RPMI 1640 medium containing 10% FCS, Hepes 10 mM and gentamicin 100 μg/ml. Supernatants were collected from the wells, cell debris removed by centrifugation and samples frozen at –80°C. IL-8 levels in the supernatants were measured by ELISA (Abnova KA0115) with sensitivity <2 pg/ml. Infections were performed in duplicate and at least twice (*n* ≥ 4). Results are expressed as IL-8 pg/ml.

### Scanning electron microscopy (SEM)

A549 cells were seeded on glass coverslips and infected as described above, by using bacteria previously grown on chocolate agar. Coverslips were fixed in ice-cold 3% glutaraldehyde for 20 min at 4°C. Samples were dehydrated in a series of graded acetone, dried by the critical point method, coated with gold in a Fine coat ion sputter JFC-1100 226 (JEOL, Ltd) and observed with an Inspect S microscope (FEI Company) working at 15 or 20 kV (Lazaro-Diez et al., 2016).

### NTHi mouse lung infection

A CD1 mouse model of NTHi lung infection was used, as described previously (Morey et al., 2013; Euba et al., 2015a,b,c). CD1 female mice (18–20 g) aged 4–5 weeks were purchased from Charles River Laboratories (France), housed under pathogen-free conditions at the Institute of Agrobiotechnology facilities (registration number ES/31-2016-000002-CR-SU-US), and used at 22–25 g. Animal handling and procedures were in accordance with the current European (Directive 86/609/EEC) and National (Real Decreto 53/2013) legislations, following the FELASA and ARRIVE guidelines, and with the approval of the Universidad Pública de Navarra (UPNa) Animal Experimentation Committee (Comité de Ética, Experimentación Animal y Bioseguridad) and the local Government authorization. NTHi375 and NTHi375Δ*thyA* were used for lung infection, and mice were randomly divided into two groups (*n* = 5): (i) control, vehicle solution (0.1 ml PBS) administered by oroesophageal gavage (Popper & Sons Inc.); (ii) one dose of TxS each 6 h administered by oroesophageal gavage, starting at 6 h post-infection (hpi). TxS (Septrin, 8 mg/40 mg/ml TMP:SMX) treatment was performed at a dose of 960 mg/kg of body weight in 0.1 ml PBS. Infecting bacteria were previously grown on chocolate agar, in the absence or presence of thymidine. For NTHi intranasal infection, 40 μl of a NTHi suspension containing ~5 × 10^8^ c.f.u./ml (~2 × 10^7^ c.f.u./mouse) were placed at the entrance of the nostrils until complete inhalation, in mice previously anesthetized with ketamine-xylazine (3:1). At 12, 24, or 48 hpi, mice were euthanized using cervical dislocation. BALF samples were obtained by perfusion and collection of 0.7 ml of PBS, with help of a sterile 20G (1.1 mm diameter) Vialon™ intravenous catheter (Becton-Dickinson) inserted into the trachea. An aliquot of each recovered BALF was serially 10-fold diluted in PBS, and plated on sBHI agar to determine the number of viable bacteria. Results are expressed as mean ± *SD* of individual log_10_ c.f.u./BALF. In parallel, lungs were removed; the left one was processed for viable bacterial counts (as detailed above), and the right lung was fixed in 10% neutral buffered formalin for histological purposes. Heads and necks containing upper airways, larynx, and tracheas were fixed in the same buffered formalin for histology. Uninfected mice receiving PBS or TxS were used as controls when necessary.

For cell counting, the remaining volume of each BALF sample was centrifuged at 5,000 r.p.m. for 3 min at 4°C. Each pellet was resuspended in 1 ml RPMI 1640 with 10% FCS and Hepes 10 mM, and total cell count determined using a hemocytometer. ~5x10^4^ cells in 200 μl RPMI 1640 with 10% FCS and Hepes 10 mM were used for cytospin preparation (1,500 r.p.m. for 10 min at room temperature, Thermo Shandon Cytospin). Giemsa stains were performed with an automated hematology slide preparation unit (SP-10, Sysmex Cosporation) according to the manufacturer's instructions. Preparations were examined in a double-blinded manner with an optical microscope (BX, Olympus).

### Histopathology and lesion score

Heads and necks were rinsed in running tap water for 1 h, immersed in 5% nitric acid for 24–36 h until complete decalcification, and 7–8 transaxial slices were made every 3–4 mm beginning at the nostrils and finishing in the caudal tracheas. Transaxial slices and lungs were embedded in paraffin, and 4–6 μm sections were stained with hematoxylin and eosin (H&E) by standard procedures, and examined by microscopy to determine the presence and extent of inflammatory lesions. Sections were examined blind as sets by a trained veterinary pathologist (Dr. M. Barberán). Parameters characterizing an acute inflammatory reaction in upper airways, larynx, trachea, and lung, including hemorrhages, hyperemia, polymorphonuclear cell infiltrates (PMNs), and alveolar macrophages, were subjectively scored on a scale of 0–3 (0: absent, 1: mild, 2: moderate, 3: severe). For tissue control, similar organs obtained from non-infected control and TxS treated mice were processed in an identical manner to the infected tissues.

### Statistical analysis

For self-aggregation, gene expression, diameter of growth or inhibition zones, PCho level, complement deposition, cell infection, bacterial loads in lungs and BALF samples, and inflammatory cells in BALF samples, mean ± *SD* were calculated and statistical comparison of means performed using the two-tail *t*-test. For histopathology scoring, means ± *SD* were also calculated and statistical comparisons performed using one-way analysis of variance (ANOVA) followed by Fisher's protected least significant difference (PLSD) multiple-comparison test. In all cases, a *p* < 0.05 value was considered statistically significant. Analyzes were performed using Prism software, version 6 for Mac (GraphPad Software) statistical package.

## Results

### Isolation of a thymidine dependent NTHi strain from the sputum of a COPD patient receiving TxS

**Figure 1:**
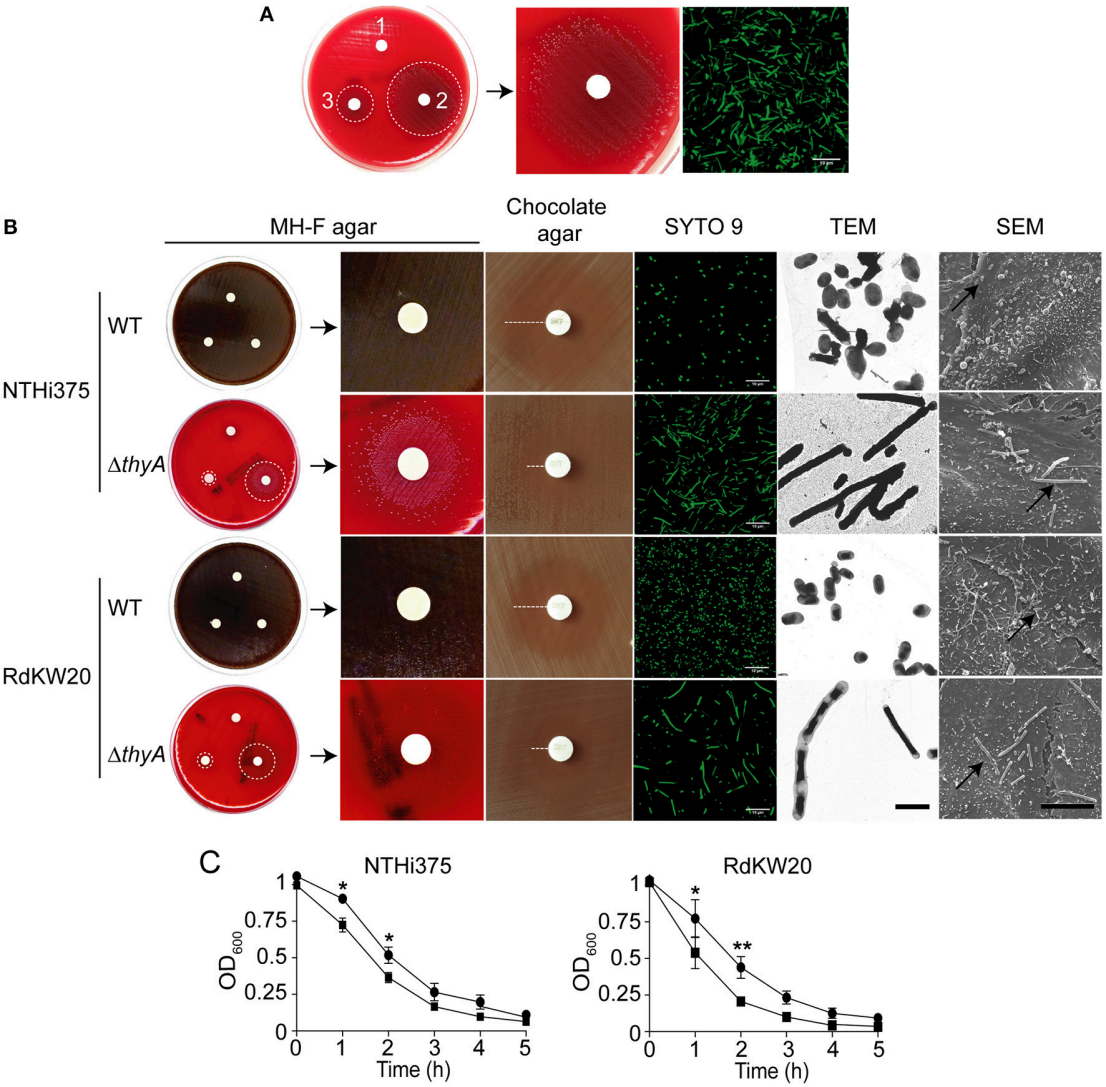
Thymidine auxotrophy modifies *H. influenzae* growth and morphology. **(A)** Growth of clinical isolate NTHi8233 on MH-F agar with sterile discs soaked in water (1), thymidine 10 mg/ml, thymidine 300 μg/ml (3). Dotted circles indicate the growth zone around discs (left). A zoom of bacterial growth around a disc soaked in thymidine 10 mg/ml is shown (middle). NTHi8233 morphology observed by SYTO 9 staining and confocal microscopy (scale bar, 10 μm; right). **(B)** Growth of NTHi375 and RdKW20 WT and Δ*thyA* strains on MH-F agar with sterile discs soaked in water (1), thymidine 10 mg/ml (2), thymidine 300 μg/ml (3). Bacterial growth rendering a lawn switches color of the MH-F agar medium from red to brown. Dotted circles indicate the growth zone around thymidine discs; zooms of bacterial growth around a disc soaked in thymidine 10 mg/ml are shown. Growth inhibition of NTHi375 and RdKW20 WT and Δ*thyA* strains on chocolate agar around TxS discs; dotted lanes indicate growth inhibition. Bacterial morphology observed by SYTO 9 staining and confocal microscopy (scale bar, 10 μm), TEM (scale bar, 2 μm; magnification 25000×), and SEM (scale bar, 10 μm; magnification 6000×). In SEM panels, black arrows indicate infecting bacteria. **(C)** Effect of thymidine auxotrophy on *H. influenzae* self-aggregation. Tube-settling experiment of bacterial cultures previously grown for 11 h on sBHI, after incubation at room temperature for 5 h. Left, NTHi375 WT and Δ*thyA* strains; right, RdKW20 WT and Δ*thyA* strains; WT strains, black circle; Δ*thyA* mutants, black square. Bacterial aggregation was quantified by measuring the decrease of absorbance at OD_600_. The *thyA* mutants self-aggregated faster than their isogenic WT strains (for NTHi375, *p* < 0.05 at 1 and 2 h; for RdKW20, *p* < 0.05 at 1 h, and *p* < 0.0005 at 2 h). All significant differences are indicated with ^*^.

Among 2,542 clinical NTHi isolated between 2010 and 2014 at Bellvitge University Hospital (Spain), 119 (4.7%) isolates formed slow-growing colonies on MH-F agar. Thymidine auxotrophy was screened on the 119 isolates by comparative growth on chocolate agar and on low-thymidine MH-F agar. Thymidine auxotrophy was confirmed by absence of bacterial growth on MH-F agar, and further growth on MH-F agar in the presence of discs soaked in thymidine. One thymidine auxotroph NTHi isolate, named strain 8233, was identified in a sputum sample recovered from a COPD patient who had received TxS for 36 days to treat an acute exacerbation by *Stenotrophomonas maltophilia*. NTHi8233 produced small colonies on chocolate agar (data not shown) and did not grow on MH-F agar, except when discs soaked with thymidine 300 μg/ml or 10 mg/ml were added to the plate. Bacterial growth showed to be dependent on thymidine concentration (Figure 1A). The thymidylate synthase encoding gene *thyA*_NTHi8233_ was sequenced, and a 6 nt duplication 267GAAAAT rendering a two-amino acid insertion (E90N91) was found, when compared to that of the RdKW20 genome sequenced reference strain (Fleischmann et al., 1995). A prediction of ThyA_*H. influenzae*_ tertiary structure, based on *Burkholderia thailandensis* ThyA protein (PDB: 3V8H), was generated by the automated modeling tool of the Swiss Model web service (http://swissmodel.expasy.org). ThyA_NTHi8233_ displayed 11 α-helices, 6 β-sheet strands and several coil connecting segments (Figure S1A). Homology between ThyA_NTHi8233_ and ThyA_RdKW20_ predicted structures is shown by structural alignment (Figure S1D).

NTHi8233 morphology was analyzed by confocal microscopy, and showed non-septated bacterial filaments ~5–10 times longer than the average NTHi bacteria (Figure 1A, right). The absence of NTHi8233 growth on MH-F agar did not allow assessing its antibiotic susceptibility following standard procedures (http://www.eucast.org/clinical_breakpoints), and TxS resistance was tested by *E*-test on bacteria grown on chocolate agar. The MIC of TxS for NTHi8233 was >32 μg/ml. NTHi8233 thymidine auxotrophy was found to be easily reversible, as described for other pathogens (Kahl, 2014). Thymidine prototroph reverted NTHi8233 strain did not present a filamented morphology (data not shown) and the sequence of the *thyA* gene did not present the 6 nt duplication described above, even though its MIC of TxS remained unchanged.

Identification of NTHi8233 supports the notion that thymidine auxotrophy may be a bacterial response to TxS treatment that could be underestimated due to the lack of growth on MH-F agar and given that, as previously stated for other NTHi isolates (Platt et al., 1983), was shown to be reversible. This observation prompted us to generate mutant strains lacking the *thyA* gene on previously characterized NTHi genetic backgrounds for a detailed study of the impact of thymidine auxotrophy for this pathogen.

### Generation and characterization of thymidine dependent *H. influenzae* strains

*H. influenzae* genome sequenced strains NTHi375 and RdKW20 were employed to generate thymidine dependent mutants by disruption of the *thyA* gene (*thyA* accession numbers NF38_0045 and HI0905, respectively). At the protein level, ThyA_NTHi375_ and ThyA_RdKW20_ displayed 98.2% identity (Figure S1E). NTHi mutants lacking the *thyA* gene were selected on sBHI agar and rendered normal size colonies (data not shown), did not grow on MH-F agar, and grew around discs soaked with thymidine on MH-F agar. Growth was dependent on the thymidine concentration present on the discs (Figure 1B and Table 2). Thus, the *thyA* mutants displayed a larger growth zone around discs soaked with thymidine 10 mg/ml than around discs soaked with thymidine 300 μg/ml (NTHi375Δ*thyA, p* < 0.005; RdKW20Δ*thyA, p* < 0.05). In contrast, wild-type (WT) bacteria presented a normal growth on MH-F agar. For NTHi375 WT and Δ*thyA* strains, the MICs of TxS were 0.25 and 0.38–0.5 μg/ml, respectively. For RdKW20 WT and *thyA* mutant strains, the MICs of TxS were 0.12 and 0.5–0.75 μg/ml, respectively. For purpose of illustration, larger growth inhibition zones were observed for WT than for *thyA* mutant strains on chocolate agar around TxS discs (Figure 1B). The morphology of NTHi375Δ*thyA* and RdKW20Δ*thyA* strains was assessed by confocal microscopy, showing non-septated filaments longer than their respective WT bacteria. Bacterial median length, measured by TEM in ~70 bacteria per strain, was (i) 1.42 ± 0.56 and 3.78 ± 2.86 μm for NTHi375 WT and Δ*thyA* strains, respectively (*p* < 0.0001); (ii) 1.43 ± 0.24 and 4.6 ± 4.98 μm for RdKW20 WT and Δ*thyA* strains, respectively (*p* < 0.0001). In agreement, SEM showed enlarged thymidine dependent bacteria on the surface of infected A549 human airway epithelial cells, which may modify the infectious process, compared to that shown by the WT strains (Figure 1B).

**Table 2:** Bacterial growth in different media and conditions.

***H. influenzae* strain**	**Chocolate agar**	**MH-F agar**	**Diameter of bacterial growth on MH-F agar around Thy^a^ disc (cm)**	
			**Thy 300 μg/ml**	**Thy 10 mg/ml**
NTHi375	Yes	Yes	Yes	Yes
NTHi375Δ*thyA*	Yes	No^b^	0.65 ± 0.05	1.83 ± 0.025
NTHi375Δ*thyA*+Thy^c^	Yes	No	1.25 ± 0.05	2.9 ± 0.1
RdKW20	Yes	Yes	Yes	Yes
RdKW20Δ*thyA*	Yes	No	0.9 ± 0.1	1.83 ± 0.08
RdKW20Δ*thyA*+Thy	Yes	No	1.4 ± 0.1	3.35 ± 0.05

a *Thy, thymidine*.

b *Total absence of bacterial growth*.

c *NTHi375ΔthyA+Thy, bacteria previously grown on chocolate+Thy were used to inoculate chocolate agar or MH-F agar plates*.

NTHi self-aggregates, which may promote microcolony formation on host cell surfaces (Meng et al., 2011; Mell et al., 2016). We asked whether the observed thymidine auxotrophy-driven increased bacterial size differs in its self-aggregation, by using tube-settling assays and monitoring the optical density of bacterial suspensions over time. Thymidine dependent mutants self-aggregated faster than their respective WT strains (Figure 1C).

Together, inactivation of the *thyA* gene in NTHi (i) impaired bacterial growth on MH-F agar, compensated by addition of external thymidine, (ii) triggered enlarged bacilli which self-aggregate faster than their isogenic WT strains, (iii) increased resistance to TxS.

### Thymidine auxotrophy causes growth defects in *H. influenzae*

**Figure 2:**
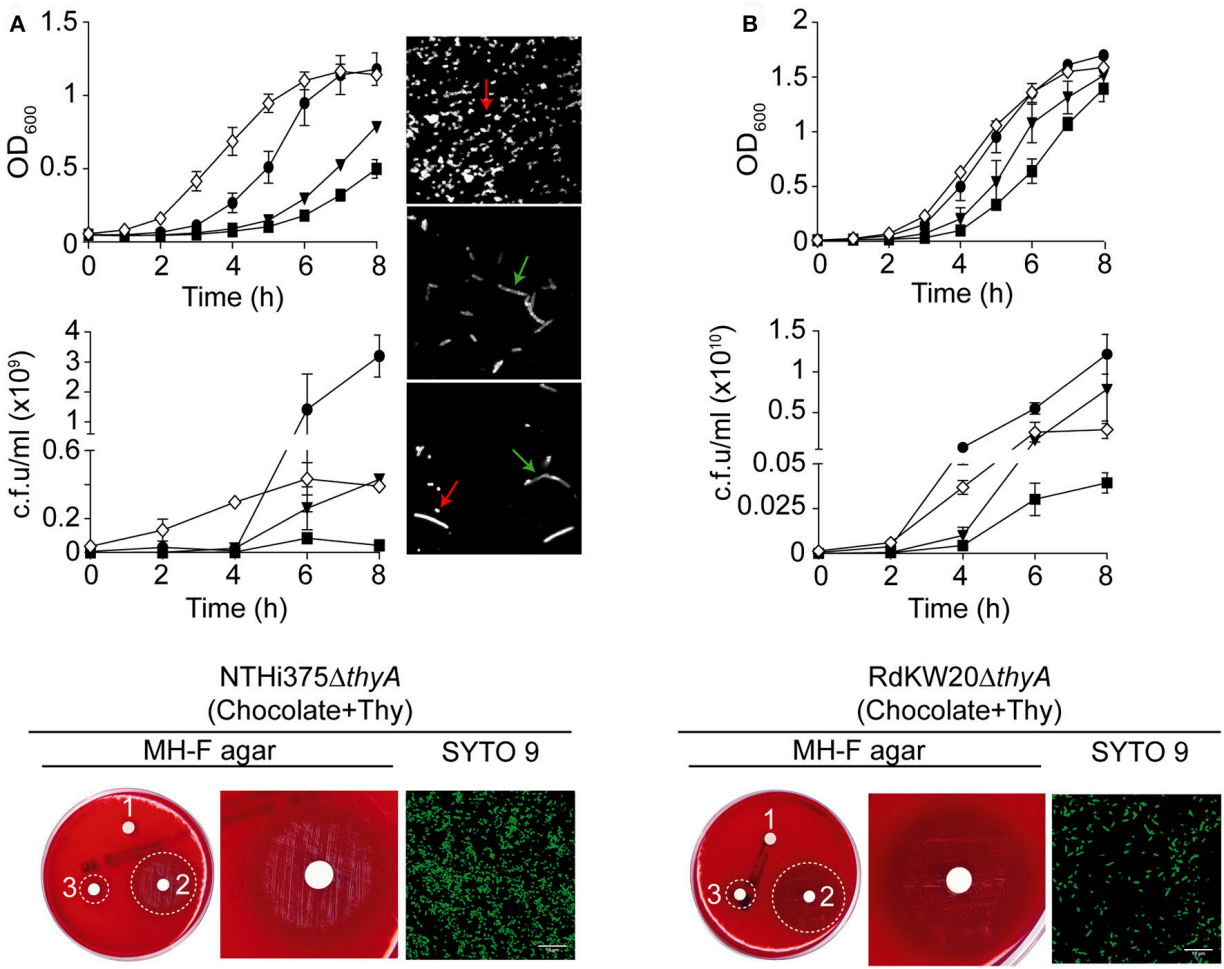
*H. influenzae* growth is modified by thymidine availability. Bacterial growth, in the absence or presence of supplemented thymidine is shown for NTHi375 **(A)** and RdKW20 **(B)** WT and Δ*thyA* strains. Growth in sBHI is shown as a means of OD_600_ (upper) and c.f.u./ml (middle) at the indicated time points. WT strains were grown in sBHI (black circle); Δ*thyA* strains were (i) grown in sBHI (black square), (ii) grown in sBHI+thymidine 300 μg/ml (inverted black triangle), (iii) pre-grown in sBHI+thymidine 300 μg/ml and further diluted in sBHI for growth recording (white diamond). **(A,B)** bottom: growth of Δ*thyA* mutants, pre-grown on chocolate agar with thymidine 300 μg/ml (chocolate+Thy) and inoculated on MH-F agar with sterile discs soaked in water (1), thymidine 10 mg/ml (2), thymidine 300 μg/ml (3). Dotted circles indicate the growth zone around discs; bacterial morphology is shown by SYTO 9 staining and confocal microscopy (scale bar, 10 μm). Immunofluorescence microscopy at the final time point of the growth curve is shown for NTHi375 WT, Δ*thyA*, and Δ*thyA* pre-grown in sBHI supplemented with thymidine 300 μg/ml (top, middle, and bottom images in **A**, right, respectively). Red arrows indicate the average NTHi WT size; green arrows indicate non-septated bacterial filaments. Bacteria were labeled with rabbit anti-NTHi primary—and a donkey anti-rabbit conjugated to Cy2 secondary antibodies.

Growth in sBHI was analyzed for WT and *thyA* mutant strains. NTHi375Δ*thyA* had an extended lag phase and lower final OD_600_ than the WT strain, which correlated with a reduced viability, measured as c.f.u./ml at the indicated time points (Figure 2A). Although not as pronounced, RdKW20Δ*thyA* also had a slightly extended lag phase and lower numbers of viable bacteria than those of the WT strain (Figure 2B). In both *thyA* mutants, the observed growth defects were partially restored by sBHI supplementation with thymidine 300 μg/ml, independently of its addition in the pre-culture used for further dilution in sBHI and OD_600_ recording, or in the actual sBHI culture used for growth monitoring over time (Figures 2A,B). Immunofluorescence microscopy at the final time point of the growth curve showed non-septated long bacterial filaments for NTHi375Δ*thyA* grown in sBHI, but a mixture of non-septated filaments and bacteria with the average NTHi WT size for NTHi375Δ*thyA* pre-grown in sBHI supplemented with thymidine 300 μg/ml (Figure 2A, right). This could relate to the observed restoration of final OD_600_ but not of final bacterial counts for NTHi375Δ*thyA* when pre-cultured in sBHI supplemented with thymidine.

The *thyA* mutants, previously grown on chocolate agar with thymidine (chocolate+Thy), grew on MH-F agar around discs in a thymidine dependent manner, displaying a larger growth zone around discs soaked with thymidine 10 mg/ml than with thymidine 300 μg/ml (NTHi375Δ*thyA, p* < 0.005; RdKW20Δ*thyA, p* < 0.005). Of note, *thyA* mutants previously grown on chocolate+Thy rendered a better growth on MH-F agar around discs soaked in thymidine than that of the same strains previously grown on chocolate agar (for thymidine 300 μg/ml, NTHi375Δ*thyA, p* < 0.05; for thymidine 10 mg/ml, NTHi375Δ*thyA, p* < 0.05, RdKW20Δ*thyA, p* < 0.005; Figures 1B, 2 bottom and Table 2). Similarly, external thymidine (chocolate+Thy) rendered *thyA* bacterial length and morphology similar to those shown by their respective isogenic WT strains (Figures 1B, 2 bottom).

### Thymidine auxotrophy modifies the expression of the nucleoside transporter encoding *nupC* gene in *H. influenzae*

Both *thyA* mutants growth on MH-F agar around discs soaked in thymidine, and morphology/growth restoration in the presence of external thymidine, prompted us to speculate that these mutants may use external thymidine to overcome their nucleoside dependency. It has been previously shown that the nucleoside transporter encoding gene *nupC* is overexpressed in *S. aureus* thymidine dependent-SCVs, and that a Δ*nupC* mutant fails to use external thymidine for growth under TxS challenge (Chatterjee et al., 2008; Kriegeskorte et al., 2014). HI0519 and NF38_02480 are annotated as *nupC* in the RdKW20 and NTHi375 genomes, respectively (Fleischmann et al., 1995; Mell et al., 2014). Expression of the *nupC* gene in *H. influenzae* was assessed by RT-qPCR, showing a trend to be higher in the Δ*thyA* than in their isogenic WT strains grown in sBHI, with stronger evidence in NTHi375 (Figure S2A). As shown in Figure 2, sBHI supplementation with thymidine partially restored the growth defects shown by the *thyA* mutants. Following the notion that thymidine supplementation may restore mutant-related phenotypes, expression of the *nupC* gene was analyzed in Δ*thyA* bacteria when pre-cultured in sBHI with thymidine and then grown in sBHI, or when grown in sBHI supplemented with thymidine. Unexpectedly, when mutant bacteria were pre-cultured in sBHI supplemented with thymidine 300 μg/ml and then grown in sBHI, expression of the *nupC* gene was even higher than when pre-cultured and grown in sBHI (Figure S2A). Of note, a total absence of growth inhibition was observed for *thyA* mutant strains on chocolate agar around TxS discs, when pre-grown on chocolate+Thy (data not shown).

Next, NTHi375Δ*nupC* and RdKW20Δ*nupC* strains were generated, showing growth rates comparable to those of their respective WT strains in sBHI (Figure S2B), chocolate agar and MH-F agar (data not shown). To assess the biological function of *nupC* as a pyrimidine transporter responsible for the uptake of extracellular thymidine in *H. influenzae*, we tested the *nupC* mutants for their ability to use external thymidine for growth under TxS challenge in a TxS disc diffusion assay. Inhibition zones on chocolate agar and MH-F agar were comparable for WT and Δ*nupC* strains. Growth inhibition around TxS discs was also tested on chocolate agar and MH-F agar plates supplemented with thymidine. Unfortunately, no conclusive differences were observed because, as already described, bacterial growth on plates with high concentration of thymidine may produce haze or fine growth areas (Lorian, 1996). Following this notion, we performed a search for pyrimidine transporters in all available genome sequenced *H. influenzae* strains, which did not reveal the presence of nucleoside transporters additional to NupC in this bacterial species. We also unsuccessfully attempted to generate a NTHi double mutant strain lacking both the *thyA* and *nupC* genes (data not shown), further supporting a role for NupC in the uptake of external thymidine.

Moreover, thymidylate is synthesized either by the thymidylate synthase ThyA or by the thymidine kinase TK, and inactivation of the former has been shown to result in upregulated expression of the later one in *Mycoplasma pneumoniae* (Wang et al., 2010). HI0529 and NF38_02430 are annotated as a *tdk* thymidine kinase in the RdKW20 and NTHi375 genomes, respectively (Fleischmann et al., 1995; Mell et al., 2014). Different to *nupC*, the *tdk* gene expression was shown to be similar in WT and Δ*thyA* strains grown in sBHI, independently of thymidine addition (Figure S2C).

Together, these results suggest that *nupC* may be a transporter for external thymidine in *H. influenzae*, whose increased expression could contribute to bypass the effects of TxS upon *de novo* thymidylate biosynthesis.

### Thymidine auxotrophy reduces PCho expression and C3b deposition in *H. influenzae*

**Figure 3:**
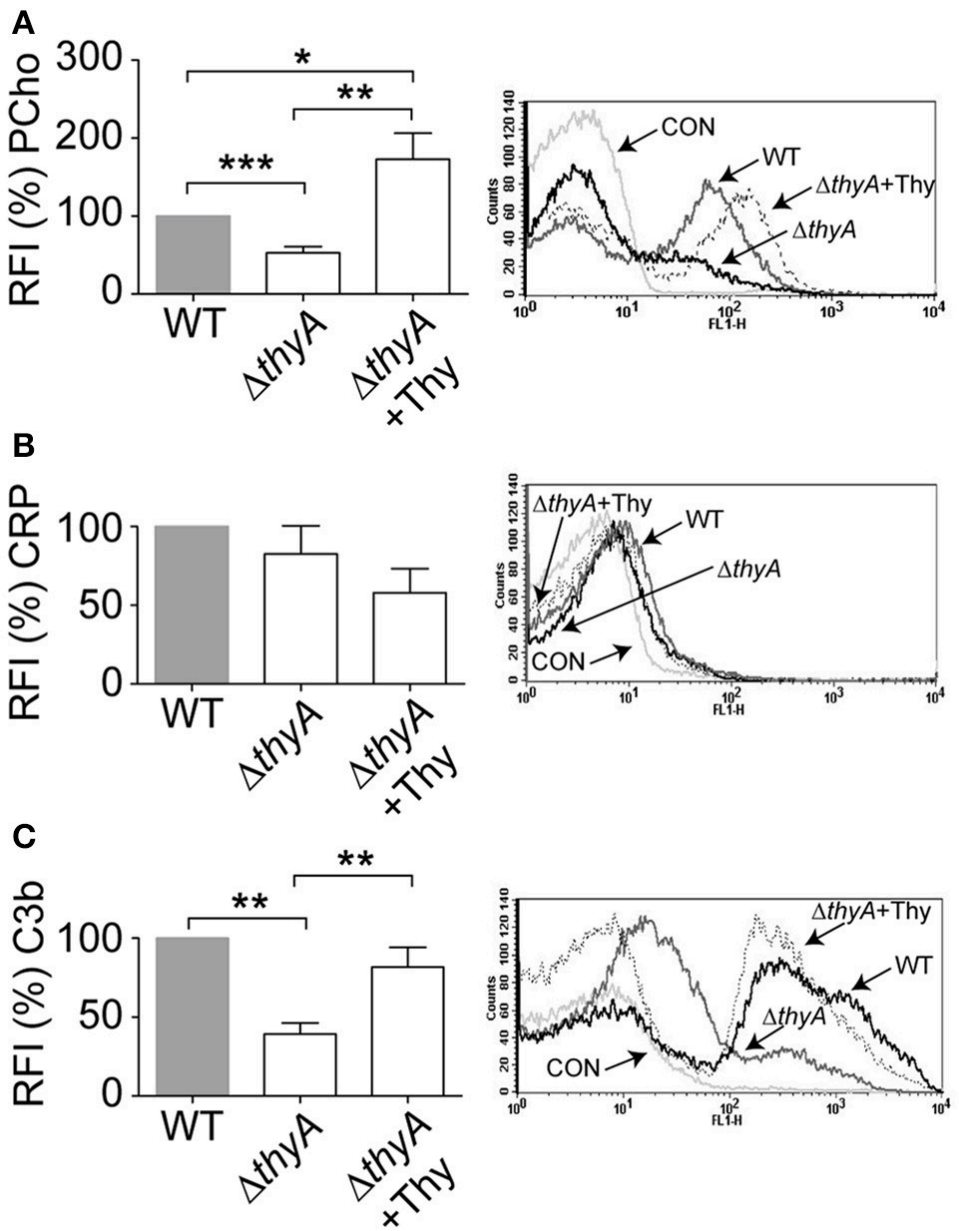
Thymidine auxotrophy reduces PCho level and C3b binding on NTHi375. NTHi375 WT and Δ*thyA* strains were exponentially grown in sBHI, in the absence or presence of thymidine 300 μg/ml, and incubated with anti-PCho **(A)**, human serum and anti-CRP **(B)** or human serum and anti-C3b **(C**) antibodies. Binding was analyzed by flow cytometry. Representative flow cytometry histograms for each assay are shown (right). Color code: light gray, control (CON) buffer; gray, NTHi375; black, NTHi375Δ*thyA*; dotted lane, NTHi375Δ*thyA* grown in sBHI+Thy. NTHi375Δ*thyA* showed significantly lower PCho level (*p* < 0.0001) and C3b deposition (*p* < 0.01) than the WT strain, which was restored by mutant bacterial growth in sBHI with thymidine 300 μg/ml (PCho, *p* < 0.01; C3b, *p* < 0.01). PCho detection was significantly higher in NTHi375Δ*thyA* grown in sBHI supplemented with thymidine than in the WT strain (*p* < 0.05). ^*^p < 0.05, ^**^p < 0.01, ^***^p < 0.0001.

We have previously reported that bacteria lacking PCho self-aggregate slightly faster (Morey et al., 2013). Thymidine auxotroph mutants showed faster self-aggregation compared to that of their WT strains (Figure 1C), which could relate to changes in cellular morphology (i.e., elongation) or, alternatively, to a different amount of PCho residues on the bacterial surface. The level of PCho was measured on NTHi375 and RdKW20 WT and *thyA* mutant strains by flow cytometry using bacteria grown in sBHI. Thymidine auxotrophy was associated with a decreased detection of PCho in both NTHi375Δ*thyA* and RdKW20Δ*thyA* mutants, which was restored in NTHi375Δ*thyA* by bacterial growth in sBHI in the presence of thymidine. Indeed, PCho detection was higher in the thymidine dependent mutants grown in sBHI supplemented with thymidine than in their isogenic WT strains (Figure 3A and Figure S3A). Decreased levels of PCho in the *thyA* mutants may modify the bacterial ability to bind C-reactive protein (CRP; Weiser et al., 1998). However, CRP deposition on the bacterial surface was similar in WT and *thyA* mutant strains (Figure 3B and Figure S3B). Finally, to assess the possibility that cellular morphology may affect complement interaction, C3b deposition was analyzed. Thymidine auxotrophy was associated with decreased detection of C3b in NTHi375Δ*thyA*, which was restored by bacterial growth in sBHI supplemented with thymidine (Figure 3C). No differences were observed for C3b deposition between RdKW20 strains (Figure S3C).

Together, inactivation of the *thyA* gene caused a reduction in the amount of PCho residues on *H. influenzae* surface, leading to impaired C3b deposition in at least NTHi375, which suggests that thymidine dependency may trigger changes affecting bacterial recognition by this key complement component by a CRP-independent mechanism.

### Thymidine auxotrophy modifies *H. influenzae* interaction with human airway epithelia

**Figure 4:**
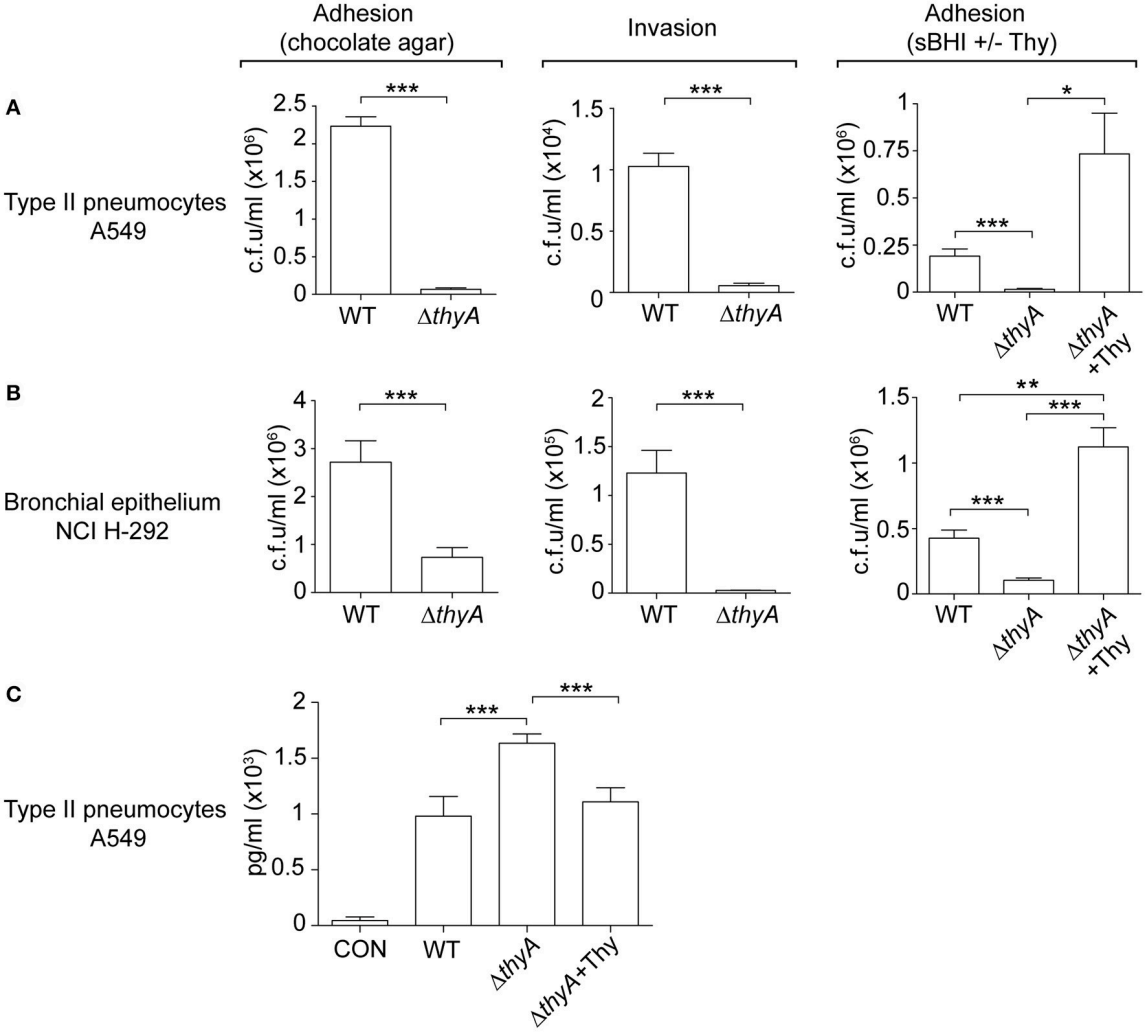
The *thyA* gene inactivation modulates *H. influenzae* interplay with cultured human airway epithelia. A549 type II pneumocytes **(A)** and NCI H-292 bronchial epithelial cells **(B)** were used to quantify adhesion and invasion by NTHi375 WT and Δ*thyA* strains. For adhesion, infecting inocula were prepared by using bacteria previously grown on chocolate agar following previously established methods (Morey et al., 2011; left). Alternatively, bacteria previously grown on sBHI, in the absence or presence of thymidine (sBHI±Thy), were used (right). NTHi375Δ*thyA* showed significantly lower adhesion to A549 (chocolate agar, *p* < 0.005; sBHI, *p* < 0.005) and NCI H-292 (chocolate agar, *p* < 0.005; sBHI, *p* < 0.005) cells than the WT strain, which was restored by mutant pre-growth in sBHI with thymidine 300 μg/ml (A549, *p* < 0.05; NCI H-292, *p* < 0.005). NTHi375Δ*thyA* showed significantly lower entry into A549 (*p* < 0.005) and NCI H-292 (*p* < 0.005) cells than the WT strain. **(C)** IL-8 release was higher in cells infected by NTHi375Δ*thyA* than by the WT strain (*p* < 0.005); NTHi375Δ*thyA* growth on chocolate+Thy reduced IL-8 secretion to WT levels (*p* < 0.005). ^*^p < 0.05, ^**^p < 0.005, ^***^p < 0.005.

The interplay of NTHi with the human respiratory epithelium plays a determinant role in the progression of infection (Clementi and Murphy, 2011). Next, we assessed if auxotrophy-related bacterial morphology changes could alter such interplay, by infecting A549 human type II pneumocytes with WT and *thyA* mutant strains (Morey et al., 2011; Lopez-Gomez et al., 2012; Euba et al., 2015c). Invasion by RdKW20 WT and *thyA* mutant strains was not assayed given the poor invasiveness of this genetic background (Mell et al., 2016). A549 cell adhesion and invasion by NTHi375Δ*thyA* was lower than that shown by the WT strain (Figure 4A). We also asked if external thymidine could restore such deficiency by using mutant bacteria grown in sBHI, in the absence or presence of thymidine (sBHI±Thy). A549 cell adhesion of NTHi375Δ*thyA* grown in sBHI was lower than that shown by the WT strain, which was restored by thymidine addition (Figure 4A, right). Similar results were obtained for RdKW20Δ*thyA* grown in sBHI, when compared to the WT strain, and to growth in sBHI with thymidine (Figure S4A). Given the impaired host cell interaction due to thymidine auxotrophy, we asked if inactivation of the *thyA* gene modifies the epithelial cell inflammatory response upon NTHi infection. Of note, the amount of secreted IL-8 was higher in A549 cells infected by NTHi375Δ*thyA* than by the WT strain, and such increase was reduced to WT levels upon infection by NTHi375Δ*thyA* previously grown on chocolate+Thy (Figure 4C). NTHi375Δ*thyA* lower epithelial infection rate was also observed during infection of NCI H-292 human bronchial epithelial cells (Euba et al., 2015c), for both adhesion and invasion (Figure 4B). NTHi375Δ*thyA* grown in sBHI showed lower adhesion to NCI H-292 cells than the WT strain, which was restored by thymidine supplementation, and adhesion by NTHi375Δ*thyA* grown in sBHI+Thy was higher than that shown by the WT strain (Figure 4B, right).

Complement opsonization, specifically C3, has been shown to enhance the bacterium-epithelial cell interaction for poorly encapsulated strains (de Astorza et al., 2004). Based on the observed differential C3b deposition by NTHi375 WT and Δ*thyA* strains, we next tested the effect of C3 in NTHi infection of A549 cells. NTHi375 and NTHi375Δ*thyA* strains were grown on chocolate agar (Δ*thyA*) or chocolate+Thy (Δ*thyA*+Thy), and A549 cells were infected in the presence of human purified C3, C3-deficient serum, or C3-deficient serum reconstituted with human purified C3. The level of bacterial attachment did not change in the presence of C3, compared to that observed in control untreated cells (CON) or cells co-incubated with C3-deficient serum (Figure S4B).

In summary, thymidine auxotrophy modified NTHi ability to infect airway epithelial cells in terms of bacterial location and triggered inflammatory response. Changes in adhesion and IL-8 secretion could be restored by addition of external thymidine in the bacterial growth medium. The presence of C3 at the onset of infection did not modulate bacterial adhesion.

### Inactivation of the *thyA* gene attenuates *H. influenzae* virulence and confers advantage under TxS treatment *in vivo*

**Figure 5:**
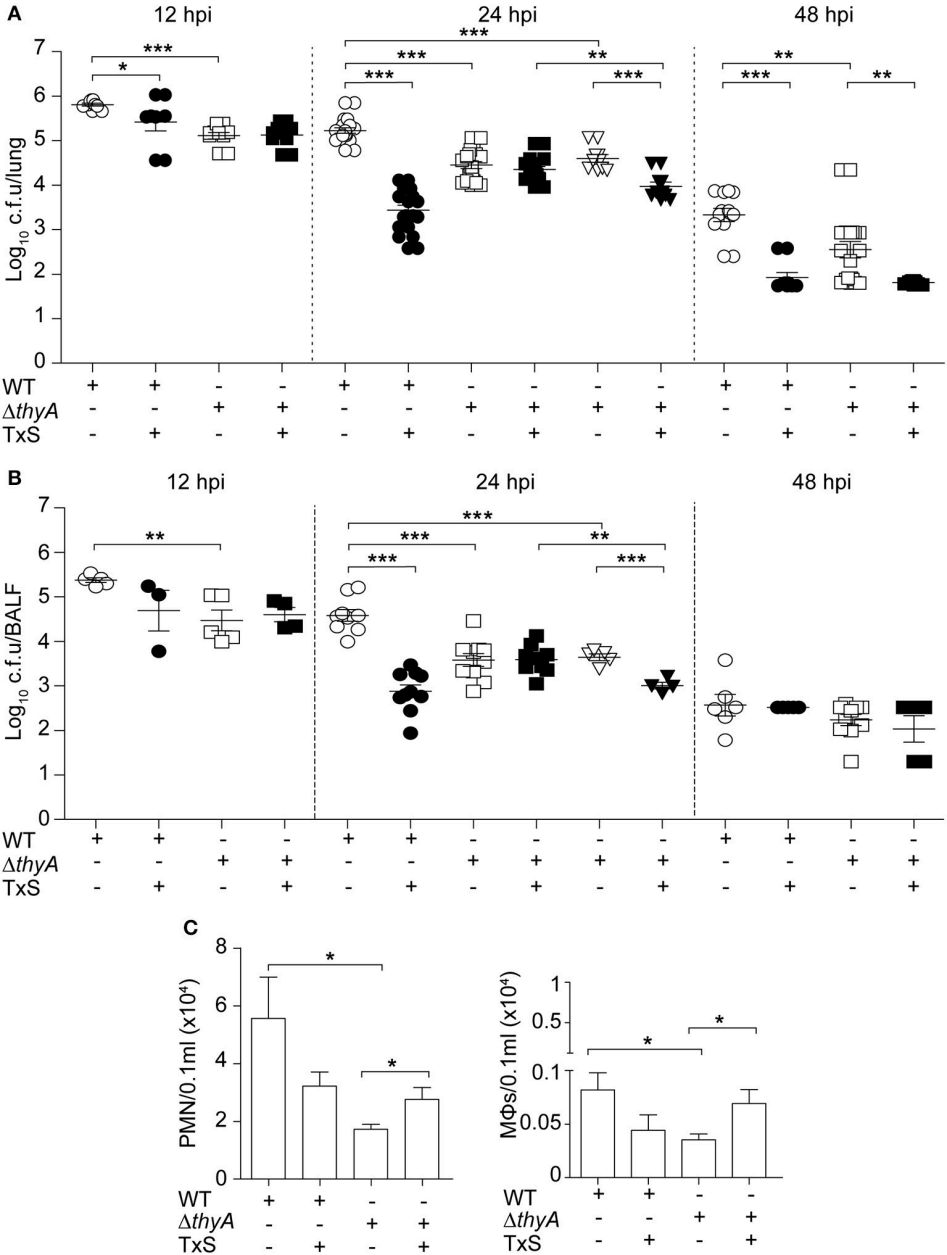
Inactivation of the *thyA* gene attenuates *H. influenzae* virulence and confers advantage under TxS treatment *in vivo*. CD1 mice were infected with ~2 × 10^7^ bacteria/mouse of NTHi375 WT (circle) or Δ*thyA* (square) strains. When necessary, a therapeutic regimen consisting of 960 mg/kg TxS (1:5 TMP:SMX) was administered orally each 6 h (dark symbols). Mice were euthanized at 12, 24, and 48 hpi, and bacterial loads were quantified in lungs (log_10_ c.f.u./lung) **(A)** and BALF (log_10_ c.f.u./BALF) **(B)**. NTHi375Δ*thyA* showed significantly lower loads in lungs and BALFs at 12 (lung, *p* < 0.001; BALF, *p* < 0.01), 24 (lung, *p* < 0.001; BALF, *p* < 0.001), and 48 (lung, *p* < 0.01) hpi than those shown by the WT strain. TxS administration significantly reduced WT strain recovery from lungs and BALF samples at 12 (lung, *p* < 0.05), 24 (lung, *p* < 0.001; BALF, *p* < 0.001), and 48 (lung, *p* < 0.001) hpi, compared to WT infected untreated mice. Inverted triangles: CD1 mice were infected with ~2 × 10^7^ bacteria/mouse of Δ*thyA* strain previously grown on chocolate+Thy for 16 h; when necessary, a therapeutic regime consisting of 960 mg/kg TxS was administered orally each 6 h (dark inverted triangles). NTHi375Δ*thyA* previously grown on chocolate+Thy rendered significantly lower loads in lungs and BALFs than the WT strain (*p* < 0.001); NTHi375Δ*thyA* previously grown on chocolate+Thy showed significantly lower loads in lungs and BALFs of TxS treated—than in those of untreated mice (*p* < 0.001); in TxS treated mice, NTHi375Δ*thyA* previously grown on chocolate+Thy showed lower loads in lungs and BALFs than NTHi375Δ*thyA* previously grown on chocolate agar (*p* < 0.01). **(C)** BALF samples were analyzed for PMN (left) and alveolar macrophage (MΦ; right) counts at 12 hpi. Significantly higher numbers were observed in NTHi375 WT than in Δ*thyA* infected samples for both PMN (*p* < 0.05) and MΦs (*p* < 0.05). ^*^p < 0.05, ^**^p < 0.01, ^***^p < 0.001.

Finally, we sought to determine the impact of thymidine auxotrophy *in vivo*, by using a mouse NTHi respiratory infection model system previously used for NTHi375 (Morey et al., 2013; Euba et al., 2015a,b). Mice were infected with NTHi375 WT and *thyA* mutant strains grown on chocolate agar, and bacterial loads were quantified in lungs and BALF samples at 12, 24, and 48 hpi. In lungs, NTHi375Δ*thyA* bacterial numbers were lower than those recovered for the WT strain at the three infection time points tested. Thymidine dependency was also associated with significantly reduced bacterial counts in BALF at 12 and 24 hpi (Figures 5A,B).

To further investigate if thymidine auxotrophy, despite attenuation, confers an advantage to *H. influenzae* under TxS treatment, we used a therapeutic regimen consisting of one TxS dose (960 mg/kg, 1:5 TMP:SMX) every 6 h, starting at 6 hpi. Results indicate that TxS treatment was more efficient in reducing bacterial counts of the WT than of the *thyA* mutant strain. Thus, a significant reduction of the WT population in lungs at 12, 24, and 48 hpi, and BALF samples at 24 hpi, was observed under treated—compared to that of untreated conditions. In contrast, bacterial counts of NTHi375Δ*thyA* were unchanged at 12 and 24 hpi in both lungs and BALFs, despite TxS administration. The protective effect of *thyA* mutation against TxS was not observed at 48 hpi, maybe due to NTHi375Δ*thyA* severe clearance at this post-infection time point.

Based on the differences observed at 24 hpi, we attempted to restore the *thyA* mutant phenotypes by infecting with mutant bacteria previously grown in the presence of thymidine. However, intranasal inoculation of NTHi375Δ*thyA* grown on chocolate+Thy did not restore bacterial counts, and lung and BALF counts for NTHi375 WT were higher than those obtained for Δ*thyA* grown on chocolate+Thy. Unexpectedly, NTHi375Δ*thyA* previous growth on chocolate+Thy increased TxS efficiency, i.e., in TxS treated animals, lung, and BALF counts were higher for NTHi375Δ*thyA* grown on chocolate agar than for NTHi375Δ*thyA* grown on chocolate+Thy. Also, lung and BALF counts for NTHi375Δ*thyA* grown on chocolate+Thy were higher in untreated—than in TxS treated animals (Figures 5A,B).

Microscopy score of the average histopathological lesion in samples of mice infected with WT or Δ*thyA* strains was also determined along the respiratory tract, and compared for untreated and TxS-treated mice (Table 3). Histopathological analysis of upper airways, larynxes, tracheas, and lungs from mice intranasally infected with WT or with Δ*thyA* rendered inflammatory lesions. Lungs and airways from control mice instilled with PBS did not show significant inflammation or pathological changes, independently of TxS administration. In both WT and Δ*thyA*-infected mice, the upper airways showed PMN infiltration of the lamina propria and lumens. PMN infiltration was found in larger numbers in the lumens of Δ*thyA* infected mice than in those infected with WT bacteria, at both 12 and 24 hpi. Conversely, PMN infiltration of the upper airway lamina propria was found to be larger in WT—than in Δ*thyA* infected mice at 24 hpi. Mild PMN infiltration of the lamina propria and lumens containing red blood cells and PMNs were the main findings in larynxes and tracheas. A tendency towards increased red blood cells containing lumens in WT-infected compared to Δ*thyA* infected mice was apparent at 24 hpi, although the differences did not reach statistical significance. As previously described (Euba et al., 2015b), lungs in infected mice showed areas of acute bronchopneumonia where alveolar septa were thickened with edema and hyperemic septal capillaries. Neutrophils, alveolar macrophages and scattered small hemorrhages were observed in alveolar spaces. Comparison of scored lesions at 12 hpi showed significantly more hemorrhages at the lower airway of mice infected with WT than at those infected with Δ*thyA* bacteria in control untreated mice. Hemorrhages were also found in larger proportions at the alveoli of mice infected with NTHi375 at 12 than at 24 hpi, but a lower proportion of alveolar macrophages was found at the lower airway of mice infected with NTHi375 at 12 than at 24 hpi. Significant differences were not found at the alveoli of Δ*thyA* infected mice between post-infection time points. Analysis of scored lesions in the airways of WT infected mice showed differences between control untreated- and TxS treated-mice. We observed a higher proportion of PMN infiltration in (i) the upper airway lumens at 24 hpi in TxS treated—than in control untreated mice, and (ii) the alveoli in control untreated—than in TxS treated mice at 12 hpi. Conversely, Δ*thyA* infection caused comparable lesions in both untreated- and TxS treated-mice (Table 3). Intranasal inoculation of NTHi375Δ*thyA* previously grown on chocolate+Thy rendered similar inflammatory lesions in both untreated- and TxS treated-animals; as a sole difference, a higher proportion of PMN infiltration was observed in the alveoli of TxS treated—than of control untreated mice (*p* < 0.05).

**Table 3:** Score of histopathological lesions found in the airways of control untreated or TxS treated mice, intranasally infected with NTHi375 WT or Δ*thyA* strains.

		**Score (mean** ± ***SD*****)^b^**								
		**Upper airways**			**Larynx-trachea**		**Lung**			
**Strain**	**Treatment^a^/hpi**	**PMNs^c^ lumen**	**PMNs lamina propria**	**Hyperemia**	**Red blood cells lumen**	**PMNs lamina propria**	**Hyperemia**	**Hemorrhage**	**Bronchial-alveolar PMNs**	**Alveolar macrophages**
NTHi375	Control/12 h	^d^^,^ ^f^1.5 ± 0.9	1.2 ± 0.4	0.8 ± 0.2	1.1 ± 1.3	0.8 ± 0.4	1.3 ± 0.3	^f^^,^ ^i^0.7 ± 0.3	^h^2 ± 0.5	^j^0.7 ± 0.2
	TxS/12 h	1.1 ± 0.6	1	1.1 ± 0.4	0.5 ± 1	0.7 ± 0.3	1.5 ± 0.7	0.3 ± 0.5	^h^1.3 ± 0.2	0.5 ± 0.4
	Control/24 h	^d^^,^ ^g^0.6 ± 0.6	^e^1.1 ± 0.3	1.1 ± 0.2	1.4 ± 0.9	0.7 ± 0.2	1.7 ± 0.6	^i^0.2 ± 0.2	1.8 ± 0.4	^j^1.4 ± 0.3
	TxS/24 h	^g^2.1 ± 1.9	1 ± 0.5	1.1 ± 0.5	1.7 ± 1.2	0.4 ± 0.2	1.7 ± 0.4	0.6 ± 0.6	1.8 ± 0.5	1.7 ± 0.2
NTHi375Δ*thyA*	Control/12 h	^d^2.5 ± 0.3	0.9 ± 0.2	0.9 ± 0.2	1.5 ± 0.8	0.7 ± 0.3	1.1 ± 0.4	^f^0.3 ± 0.2	1.7 ± 0.2	0.4 ± 0.2
	TxS/12 h	2.1 ± 0.4	0.9 ± 0.2	0.6 ± 0.2	0.8 ± 1.1	0.8 ± 0.4	0.9 ± 0.4	0.1 ± 0.2	1.7 ± 0.5	0.8 ± 0.4
	Control/24 h	^d^1.7 ± 1.1	^e^0.6 ± 0.2	1 ± 0.3	0.5 ± 0.8	0.7 ± 0.2	1.6 ± 0.4	0.2 ± 0.2	2 ± 0.3	1.1 ± 0.4
	TxS/24 h	1.8 ± 0.2	0.8 ± 0.2	0.8 ± 0.3	0.5 ± 1	0.6 ± 0.4	1 ± 0.4	0.1 ± 0.2	1.9 ± 0.4	1.1 ± 0.2

a *Control, animals administered vehicle solution; TxS, postinfection, one TxS dose was administered each 6 h*.

b *Statistical comparisons of mean values were performed using one-way ANOVA followed by Fisher's PLSD multiple-comparison test*.

c *PMNs: infiltrates of polymorphonuclear cells*.

d *More recruitment of PMNs at the upper airway lumen of mice infected with NTHi375ΔthyA than with WT at 12 and 24 hpi (both, p < 0.05)*.

e *More recruitment of PMNs at the upper airway lamina propria of mice infected with WT than with ΔthyA bacteria at 24 hpi (p < 0.05)*.

f *Higher alveolar hemorrhage in the lungs of mice infected with WT than with ΔthyA bacteria at 12 hpi (p < 0.05)*.

g *Lower proportion of PMNs at the upper airway lumen (24 hpi) of WT bacteria infected mice untreated than TxS treated (p < 0.05)*.

h *Larger proportion of PMNs at the alveoli (12 hpi) of WT bacteria infected mice untreated than TxS treated (p < 0.05)*.

i *Higher alveolar hemorrhage in the lungs of mice infected with WT bacteria at 12 than at 24 hpi (p < 0.01)*.

j *Lower numbers of alveolar macrophages at the alveoli of mice infected with WT bacteria at 12 than at 24 hpi (p < 0.01)*.

Last, we quantified accumulation of immune cells in the collected BALF samples at 12 hpi. NTHi375 WT pulmonary infection increased the accumulation of PMNs and alveolar macrophages, compared to that observed in the BALF of Δ*thyA* infected mice. TxS treatment showed a trend to reduce immune cell accumulation in BALF samples of WT infected mice. Unexpectedly, TxS treatment increased immune cell accumulation in BALF samples of Δ*thyA* infected mice (for both PMNs and alveolar macrophages; Figure 5C).

In summary, thymidine auxotrophy reduces NTHi virulence but, concomitantly, it confers an advantage under TxS treatment *in vivo*, suggesting that uptake of external thymidine from the infected tissue may contribute to bypass the bactericidal effect of TxS. Moreover, overall higher inflammatory traits were observed in WT—than in Δ*thyA* infected mice.

## Discussion

First-line antimicrobial agents must be effective, reliable, widely available and affordable in resource-poor settings (Grant et al., 2009). Following this notion, oral TxS has been recommended for years as initial antibacterial for acute otitis media, non-severe pneumonia or AECOPD because of its effectiveness and reasonable price, which, in turn, has resulted in increased resistance patterns by frequent respiratory pathogens such as *H. influenzae*. Thus, *H. influenzae* increased TxS resistance has been reported in clinical isolates from serotypes b, e, and f, and non-typeable strains from various pathological origins (Rowe et al., 2000; Leiberman et al., 2001; Campos et al., 2003a,b; Arguedas et al., 2005; Mohd-Zain et al., 2012; Puig et al., 2014; Greenhill et al., 2015). Besides *H. influenzae* TxS resistance due to changes in the sequence and/or expression of the *folH* and *folP* genes, or acquisition of the *sul* genes (de Groot et al., 1988, 1996; Enne et al., 2002), TMP resistance has been reported to arise in this pathogen as an indirect result of mutation to thymidine/thymine auxotrophy (Platt et al., 1983). Standard procedures for determination of *H. influenzae* antibiotic susceptibility at diagnostic laboratories involve the use of the low thymidine containing medium MH-F, which could underestimate the frequency of thymidine-dependent TMP/TxS resistance. Indeed, the routine evaluation of NTHi clinical isolates carried out in this study reported a proportion of strains designated as forming slow-growing colonies, which prompted us to screen their thymidine dependency, leading to identification of the thymidine auxotroph NTHi8233. Compared to ThyA_RdKW20_, ThyA_NTHi8233_ has a two-amino acid insertion at positions 90 and 91, which slightly modifies its predicted structure (Figure S1). Inactive *thyA* gene alleles have been reported for *S. aureus* due to in-frame deletions, deletions resulting in a frameshift, or point mutations resulting in amino acid transitions or non-sense mutations (Chatterjee et al., 2008; Kriegeskorte et al., 2014). In this study, we identified a NTHi *thyA* allele with an in-frame insertion compared to previously genome sequenced strains, which could alter its enzymatic activity. The ThyA canonical active site tryptophan (W83) proposed for bacterial sequences (Baugh et al., 2013) is conserved in ThyA_NTHi8233_, and future work will attempt to purify ThyA_NTHi8233_ and further assess its thymidylate synthase activity.

Moreover, NTHi8233 thymidine auxotrophy was found to be reversible, and such reversion was associated to disappearance of the in-frame insertion in the *thyA*_NTHi8233_ gene. This transient phenotype, in agreement with previous observations (Platt et al., 1983), prompts us to speculate that reversible thymidine auxotrophy may lead into the infrequent isolation of thymidine dependent NTHi strains from respiratory samples. In contrast, TxS resistance level remained unchanged in NTHi8233, independent of auxotrophy reversion. Of note, increased TMP resistance has been previously found in *H. influenzae* thymidine prototrophs isolated soon after isogenic thymidine auxotrophs recovery from sputum samples exposed to subinhibitory TMP concentrations, suggesting that TMP resistance could be the result of a suppressor mutation (Platt et al., 1983). Following this observation, the MIC of TMP for NTHi8233 was >32 μg/ml, independent of auxotrophy reversion. The mechanism(s) underlying TMP/TxS resistance in NTHi8233 is currently unknown and will be subject of future study.

Reversible thymidine auxotrophy has been widely shown for *S. aureus* thymidine dependent-SCVs (Kahl, 2014), and made NTHi8233 inadequate for analysis of the impact of thymidine dependency on NTHi pathogenesis. For this purpose, the *thyA* gene was inactivated in strains NTHi375 and RdKW20. NTHi *thyA* mutants displayed alterations in morphology and growth, and dependence on external thymidine, comparable to those shown by *S. aureus, Salmonella typhimurium*, or *S. maltophilia* thymidine auxotrophs (Kok et al., 2001; Kahl et al., 2003, 2005; Anderson et al., 2007; Chatterjee et al., 2008). Moreover, NTHi *thyA* gene disruption caused a reduced PCho level and C3b deposition, impaired airway epithelial adhesion and invasion, and enhanced secretion of IL-8 by cultured epithelial cells. In some cases, minor phenotypic differences were observed between NTHi375 and RdKW20 *thyA* mutants, likely to be related to the known NTHi genomic heterogeneity (De Chiara et al., 2014). Altogether, the observed *in vitro* phenotypes, such as a deficient interaction with—and an increased inflammatory response by cultured airway epithelial cells upon infection by Δ*thyA* mutant strains, are likely to contribute to NTHi thymidine auxotroph attenuation upon murine lung infection. Modification of the biology of infection due to thymidine auxotrophy has been reported for other bacterial pathogens including *S. aureus, S. typhimurium, Shigella flexneri*, or *Vibrio cholerae* (Attridge, 1995; Cersini et al., 1998; Kok et al., 2001; Kriegeskorte et al., 2014). We acknowledge that a limitation of this study is the lack of genetic complementation for the *thyA* gene inactivation. Several approaches were unsuccessfully undertaken for plasmid-encoded *thyA*_RdKW20_ gene complementation into RdKW20Δ*thyA*, and plasmid-encoded heterologous expression of the *thyA*_NTHi8233_ allele into RdKW20Δ*thyA* (data not shown). Of note, we exclude a relationship between the *thyA* gene-related phenotypes shown in this study and overexpression of the immediately downstream gene, a putative homolog of the *tadA* gene encoding a tRNA-specific adenosine deaminase, in the *thyA* mutants (data not shown). Overexpression of the *tadA* gene has been shown to confer resistance to the bactericidal natural product xanthorrhizol in *E. coli* (Yogiara et al., 2015). Importantly, addition of external thymidine in the growth media restored, partial or totally, all analyzed *in vitro* phenotypes. Attenuation of NTHi375Δ*thyA in vivo* was not restored by infecting bacteria previously grown in the presence of external thymidine, which could be due to the observed partial restoration of thymidine prototrophy by available external thymidine, shown to be dependent on thymidine concentration on MH-F agar.

In addition to the impact of thymidine auxotrophy by inactivation of the *thyA* gene on NTHi virulence, TxS resistance was shown to increase in the Δ*thyA* mutant strains. Disruption of the *thyA* gene has been related to increased TMP resistance in other bacteria (Song et al., 2016). In agreement, the MIC of TMP was 1 and >32 μg/ml for NTHi375 WT and Δ*thyA* strains, respectively, and was 0.75 and >32 μg/ml for RdKW20 WT and Δ*thyA* strains, respectively. NTHi375Δ*thyA* pre-grown in chocolate+Thy was cleared faster in TxS-treated than in untreated mice, and the reason(s) for this observation is currently unknown. Although, originally intended to restore the observed increased expression of the *nupC* gene upon *thyA* inactivation, mutant pre-growth in sBHI with thymidine further amplified *nupC* expression, compared to sBHI. It should be noted that increased *nupC* gene expression by a *thyA* mutant in *S. aureus* was previously restored by complementing *thyA* gene disruption (Chatterjee et al., 2008). Our observation was unexpected and formally incomparable to that made for *S. aureus*. We speculate that it could be associated with the lack of growth inhibition around TxS discs observed for *thyA* mutant strains when pre-grown in chocolate+Thy, therefore suggesting that uptake of external thymidine by the NupC nucleoside transporter could contribute to circumvent the effects of TxS upon *de novo* thymidylate biosynthesis in NTHi. We suggest here for the first time that NupC could function as a primary thymidine transporter in NTHi if *de novo* thymidylate synthesis is blocked. Search in all available genome sequenced *H. influenzae* strains did not reveal the presence of additional nucleoside transporters in this bacterial species. As expected, we unsuccessfully attempted to generate a NTHi double mutant strain lacking both the *thyA* and *nupC* genes (data not shown), further supporting the proposed role for NupC.

Three additional aspects deserve further discussion. First, *S. aureus* thymidine dependent SCVs grow on Columbia blood agar (Kriegeskorte et al., 2014); *S. maltophilia* thymidine dependent SCVs grow on sheep blood-, brucella-, and chocolate-agar (Anderson et al., 2007), but neither grow on MacConkey agar and M9 minimal medium (Anderson et al., 2007), nor *S. typhimurium thyA* mutants grow on LB agar (Kok et al., 2001). Our results show that NTHiΔ*thyA* thymidine auxotrophs grow rendering normal size colonies on chocolate—and sBHI-agar, but fail to grow on MH-F agar. Therefore, we may not consider the term thymidine dependent SCVs for *H. influenzae*. Second, *S. aureus* SCVs are recovered from several human and animal specimens, and are a highly dynamic subpopulation optimized for persistence, enabling the bacteria to hide inside the host cell without eliciting a strong host response (Kahl, 2014; Kahl et al., 2016). This may be unlikely for NTHi thymidine auxotrophs, given that we observed a significantly impaired airway epithelial cell invasion by the NTHi375Δ*thyA* mutant strain. Third, this study does not tackle the induction of NTHi thymidine auxotrophy by TxS challenge, and we therefore cannot speculate on the selection for thymine auxotrophs and TxS resistance due to prolonged antibiotic exposure. Unexpectedly, no statistically significant connection has been found between regional TxS use and resistance among *H. influenzae* isolates (Karpanoja et al., 2008). We should also consider the potential emergence of mutations in the *thyA* gene as a consequence of treatment with other antibiotics with mutagenic activity such as ciprofloxacin, which concomitantly increases TMP resistance (Song et al., 2016). Hence, emergence of TxS resistance in response to exposure to antibiotics needs further analysis and continuous monitoring.

In conclusion, this study shows for the first time the impact of thymidine auxotrophy by disruption of the thymidylate synthase *thyA* on *H. influenzae* morphology and interplay with the host airway and, indirectly, on its resistance to TxS. We also show NupC as a potential facilitator of external thymidine uptake upon inhibition of thymidylate *de novo* synthesis. Thymidine auxotrophy lowers NTHi virulence, but also provides an advantage under TxS exposure. Thus, our results should be considered for the consequences of TxS administration in the clinical settings. Further studies will contribute to better assess the emergence of NTHi thymidine auxotrophs in clinical samples, likely to be currently underestimated, and to understand if NTHi thymidine dependency may be a response to antibiotic treatment with a survival advantage in specific environments.

## Author contributions

IR, SM, BE, AF, JM, NL, MB, JR, FT, and CL have participated in the design and fulfillment of the experimental work. IR, SM, CA, JL, JY, and JG carried out in the conceptual design of the study. IR, SM, JL, JY, and JG have written the manuscript (text, tables, and figures). All authors have participated in the correction of the manuscript to its final version.

### Conflict of interest statement

The authors declare that the research was conducted in the absence of any commercial or financial relationships that could be construed as a potential conflict of interest.
