# Supplementary material for: Inactivation of the Thymidylate Synthase thyA in Non-typeable Haemophilus influenzae Modulates Antibiotic Resistance and Has a Strong Impact on Its Interplay with the Host Airways
Source: Front Cell Infect Microbiol. 2017 Jun 20;7:266. doi: 10.3389/fcimb.2017.00266 (PMC5476696; doi:10.3389/fcimb.2017.00266)
Supplement: Supplementary file 1 [file Image1.PDF]

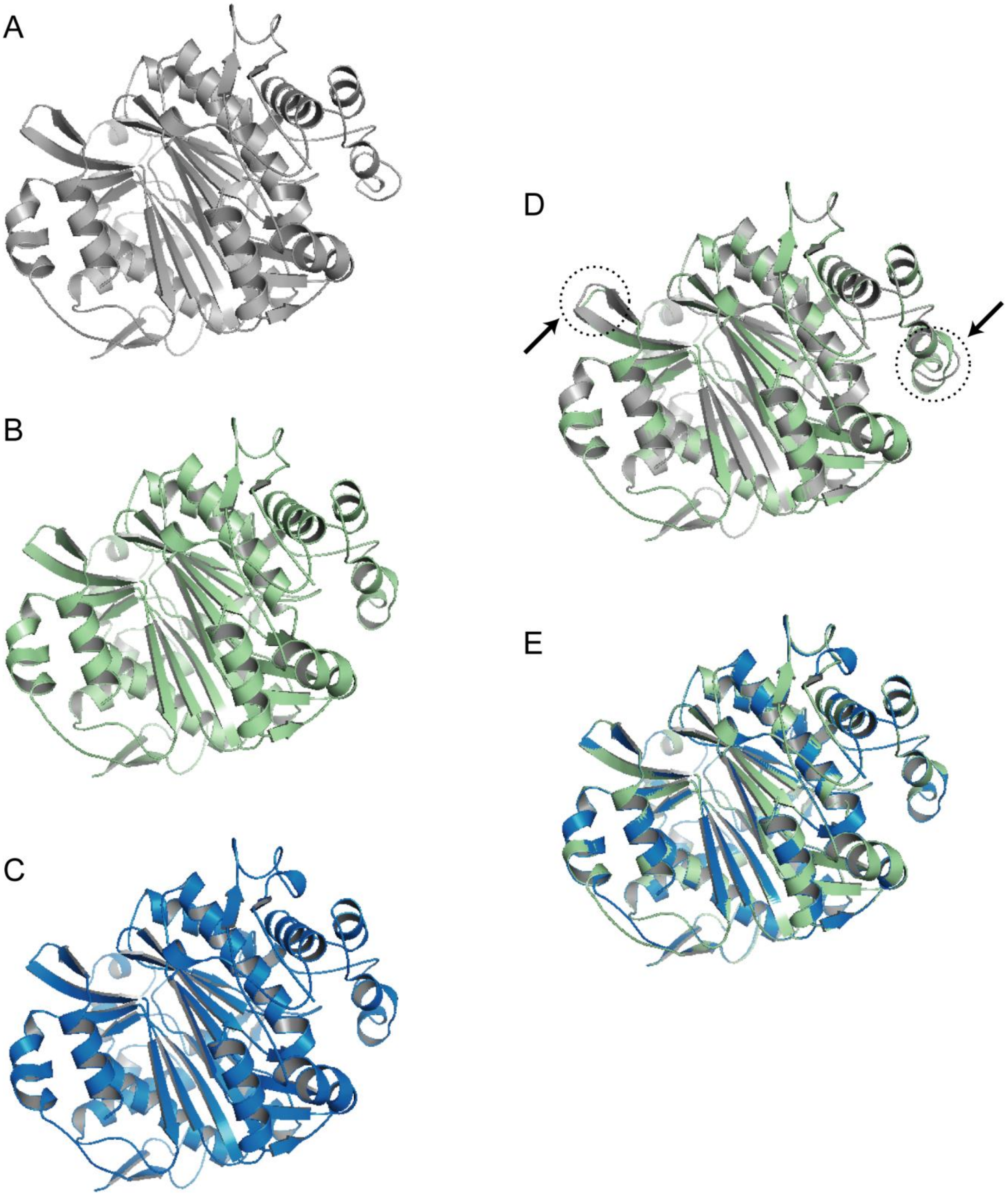

Figure S1. Predicted protein structure for ThyA$_{Hi8233}$(A), ThyA$_{RdKW20}$ (B) and ThyA$_{NTHi375}$ (C), generated by the automated modeling tool of the Swiss Model web service (http://swissmodel.expasy.org). Protein structural alignments and representation were generated with the molecular visualization software Open PyMOL (http://www.pymol.org). Superpositions of ThyA$_{NTHi8233}$ and ThyA$_{RdKW20}$ **(D)**, and of ThyA$_{RdKW20}$ and ThyA$_{NTHi375}$ proteins **(E)** are shown. Structural changes between ThyA$_{NTHi8233}$ and ThyA$_{RdKW20}$ are circled and arrow pointed. Right arrow indicates amino acids E90 and N91 in ThyA$_{NTHi8233}$ (absent in ThyA$_{RdKW20}$).

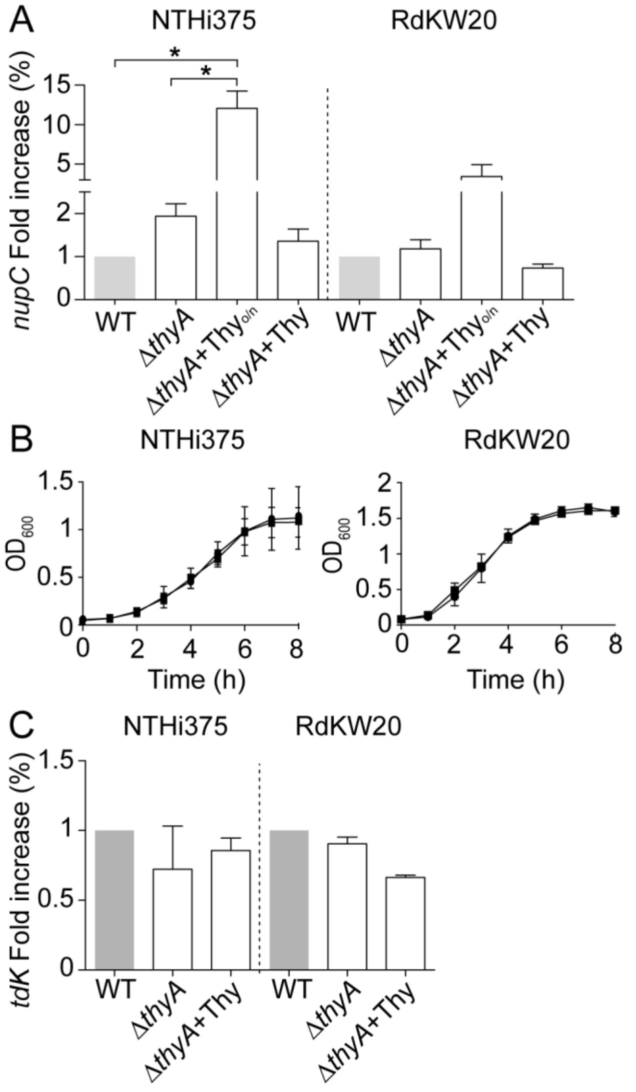

Figure S2. (A) *H. influenzae* thymidine auxothrophy modulates expression of the nucleoside transporter *nupC*. Expression of the nucleoside transporter encoding *nupC* gene upon bacterial growth in sBHI, in the absence or presence of thymidine, is shown for NTHi375 and RdKW20 WT and *thyA* strains. WT strains were grown in sBHI; Δ*thyA* strains were grown in sBHI (Δ*thyA*), sBHI+thymidine 300 µg/ml (ΔthyA+Thy), or were o/n pre-cultured in sBHI+thymidine 300µg/ml and grown in sBHI (Δ *thyA*+Thy$_{o/n}$). Expression of the *nupC* gene was higher in the *thyA* mutants pre-cultured in sBHI+thymidine (Δ *thyA*+Thy$_{o/n}$) than in the *thyA* mutants grown in sBHI (Δ*thyA*) (for NTHi375, $p<0.05$). **(B) Mutation of the *nupC* gene does not modify NTHi growth.** Bacterial growth in sBHI is shown for NTHi375 and RdKW20 WT and Δ*nupC* strains. **(C) *H. influenzae* thymidine auxothrophy does not modulate expression of the thymidine kinase *tdk*.** Expression of the thymidine kinase *tdK* gene upon bacterial growth in sBHI, in the absence or presence of thymidine, is shown for NTHi375 and RdKW20 WT and *thyA* mutant strains. WT strains were grown in sBHI; Δ*thyA* strains were grown in sBHI (Δ*thyA*) or sBHI+thymidine 300 µg/ml (Δ *thyA*+Thy)

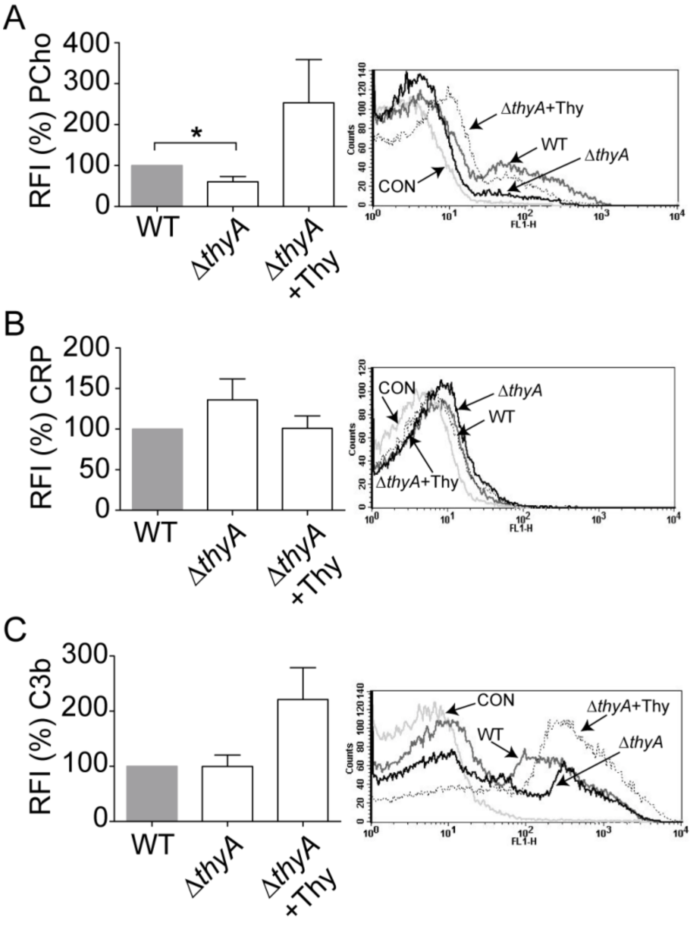

Figure S3. Thymidine auxotrophy reduces PCho level in RdW20. RdKW20 WT and Δ*thyA* strains were exponentially grown in sBHI, in the absence or presence of thymidine 300 µg/ml, and incubated with anti-PCho (A), human serum and anti-CRP (B) or human serum and anti-C3b (C) antibodies. Binding was analyzed by flow cytometry. Representative flow cytometry histograms for each assay are shown (right panels). Color code: light gray, control (CON) buffer; gray, RdKW20; black, RdKW20 Δ*thyA*; dotted lane, RdKW20Δ*thyA* grown in sBHI+Thy. RdKW20Δ*thyA* showed significantly lower PCho level ($p<0.05$) than the WT strain, which was restored by mutant bacterial growth in sBHI with thymidine 300 µg/ml.

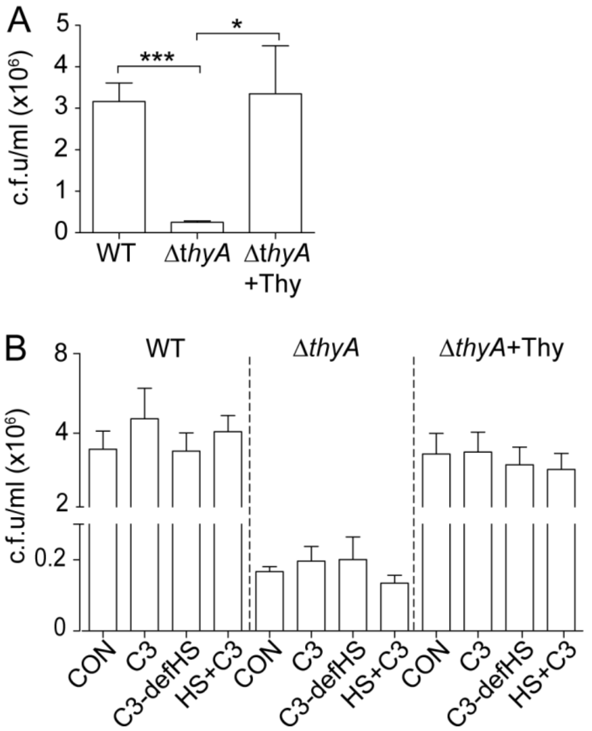

Figure S4. Modulation of *H. influenzae* cell infection by thymidine dependency and host complement. (A) Inactivation of the *thyA* gene reduces adhesion of *H. influenzae* RdKW20 to epithelial cells. A549 cells were used to quantify adhesion of RdKW20 WT and Δ*thyA* strains. Bacteria pre-grown in sBHI, in the absence or presence of thymidine 300 μg/ml, were used. NTHi375Δ*thyA* showed significantly lower adhesion to A549 cells ($p<0.0001$) than the WT strain, which was restored by mutant pre-growth in sBHI with thymidine ($p<0.05$). **(B)** NTHi375 WT and Δ*thyA* strains were grown on chocolate agar (Δ*thyA*) or chocolate+Thy (Δ*thyA*+Thy) and A549 cells were infected in the presence of human purified C3, C3-deficient serum, or C3-deficient serum reconstituted with human purified C3. Bacterial attachment remained unchanged in the tested conditions.
